# Punishment after Life: How Attitudes about Longer-than-Life Sentences Expose the Rules of Retribution

**DOI:** 10.3390/bs14090855

**Published:** 2024-09-23

**Authors:** Eyal Aharoni, Eddy Nahmias, Morris B. Hoffman, Sharlene Fernandes

**Affiliations:** 1 Department of Psychology, Georgia State University, Atlanta, GA 30302, USA; 2 Department of Philosophy, Georgia State University, Atlanta, GA 30302, USA; 3 Second Judicial District (Ret.), State of Colorado, Denver, CO 80203, USA

**Keywords:** punishment, criminal sentencing, retribution, heuristics, life sentence, judgment

## Abstract

Prison sentences that exceed the natural lifespan present a puzzle because they have no more power to deter or incapacitate than a single life sentence. In three survey experiments, we tested the extent to which participants support these longer-than-life sentences under different decision contexts. In Experiment 1, 130 undergraduates made hypothetical prison sentence-length recommendations for a serious criminal offender, warranting two sentences to be served either concurrently or consecutively. Using a nationally representative sample (N = 182) and an undergraduate pilot sample (N = 260), participants in Experiments 2 and 3 voted on a hypothetical ballot measure to either allow or prohibit the use of consecutive life sentences. Results from all experiments revealed that, compared to concurrent life sentences participants supported the use of consecutive life sentences for serious offenders. In addition, they adjusted these posthumous years in response to mitigating factors in a manner that was indistinguishable from ordinary sentences (Experiment 1), and their support for consecutive life sentencing policies persisted, regardless of the default choice and whether the policy was costly to implement (Experiments 2 and 3). These judgment patterns were most consistent with retributive punishment heuristics and have implications for sentencing policy and for theories of punishment behavior.

## 1. Introduction

After killing 23 people and injuring 22 more in El Paso, Texas, gunman Patrick Crusius was sentenced to 90 consecutive life sentences without the possibility of parole [1]. In 2019, Brenton Tarrant was sentenced to 52 life sentences plus 480 years without parole for shooting 51 people to death and injuring many others in his rampage of two mosques in Christchurch, New Zealand [2]. When it comes to killers, governments across the globe frequently punish criminals with sentences that far exceed the humaen lifespan.

The persistence of longer-than-life sentences raises an interesting question that has been overlooked in punishment research. If punishment judgments resulted from rational deliberation to optimize for the best consequences, as so-called *consequentialist* theories of jurisprudence assume [3], then people should never have a systematic preference for sentences that are longer than a human life span over true life sentences. Longer-than-life sentences do not control the offender’s behavior or protect society any better than an actual life sentence does. So, why are such sentences, which cannot be served, codified into law and so frequently imposed? One interpretation is that this practice is merely a procedural artifact of the way our legal system happens to be set up, which often imposes a mandatory minimum sentence for the most severe crimes and assigns a separate sentence for each victim. Typically, sentences are imposed concurrently, by default, but if the judge orders these sentences to be served consecutively, which is usually discretionary but in some circumstances is mandatory (e.g., [4,5,6,7]), the sentences are added together (see [8,9]). This simple addition rule was plausibly designed for offenders with multiple short-term sentences. The fact that their sum can exceed a lifespan is legally meaningless, according to this view.

But even if this procedural artifact theory is true, longer-than-life sentences might not be psychologically meaningless. First, these punishments might serve a retributive, communicative, or symbolic function (see [8,10,11,12,13]). According to this framework, one goal of punishment is to give offenders what they deserve or to express the wrongfulness of the offense to the community (including victims’ families, the offender, and potential offenders), and some offenses are more wrongful than can be expressed by a mere life sentence. (See Future Directions section for more on communicative theories.) This theory could explain why many state consecutive sentencing statutes (e.g., CA, FL, IL, and TX) specifically target the most serious offenders. Seemingly symbolic punishments can also be found in other contexts, whereby the offender’s dangerousness level is not in question, including lengthy sentences for quadriplegics [14] and centenarians [15] as well as multiple death sentences for those who murder multiple victims. In these types of situations, people might feel that regardless of the punishment’s circumstances, the offender still deserves punishment in an amount that reflects the gravity of his crimes, and that this obligation carries important symbolic value to offenders, victims, and the community. However, this prediction has never been tested with respect to sentences that extend beyond the offender’s lifespan.

Even if retributive or communicative theories accurately characterize people’s explicit reasons for punishment, the question remains of why people would feel so compelled to express, through longer-than-life sentences, the wrongfulness of the offense, given that such sentences are no more effective at incapacitating the offender (or potential offenders) than is a true life sentence. In other words, if a longer-than-life sentence is purely symbolic—such as a symbol of the lost lives of *multiple* victims—why would the punisher not be equally satisfied by a symbol that represents those multiple victims using multiple but *concurrent* life sentences instead of consecutive ones? Perhaps, as an empirical matter, longer-than-life sentences pack a more general deterrent punch than ordinary life sentences. But if so, this too demands explanation, for the same reason that longer-than-life sentences are no more consequential for the (prospective) offender than are true life sentences. These posthumous sentences carry no actual bargaining power. So the prospect that punishers feel that it is important to express the empty threat of a sentence that extends well beyond the natural lifespan—to the offender or anyone else—calls for explanation.

One possible explanation for why people would support longer-than-life sentences is that their judgments in support of such sentences are produced not by conscious reflection about the consequences but by a heuristic process. By heuristic, we mean the (often unconscious and intuitive) application of a simple rule of thumb that enables people to make decisions efficiently and with limited information and resources [16]. In the environments in which our punishment psychology might have evolved, the failure to punish (and thus incapacitate or deter) a violent perpetrator could have had dire consequences. So, under uncertainty, evolution might have favored the propensity to punish too much rather than too little [17]. This theory could explain ancestral behaviors such as killing the innocent family members of a murderous perpetrator [18]. The apparent irrationality of punishing too much could itself have advantages, for example, by advertising the punisher’s commitment to the cause [19]. Like modern justifications for punishment, evolved punishment motivations could ultimately serve to incapacitate or to communicate a message, but through an unconscious, heuristic process rather than a reflective, instrumental one [20]. Indeed, the retributive punishment justification itself has been proposed as the expression of this heuristic process [21].

The empirical evidence for the heuristic basis of moral judgments, including retributive punishment judgments, is substantial (e.g., [21,22,23,24,25,26,27]). A hallmark of heuristic cognition is that it operates relatively automatically and systematically, even if its practical function is overgeneralized, such as overestimating the likelihood of a shark attack, despite statistical evidence to the contrary (e.g., [28]). Of course, ancestral environments had no direct equivalent to longer-than-life prison sentences, so the question is whether our evolved punishment psychology overgeneralizes by applying the same rules used in real punishment judgments to the (inconsequential) posthumous range, perhaps in part because, within the constraints of the modern legal system, there is no other obvious way to signal punishment for the worst crimes (e.g., with multiple victims or multiple heinous crimes against one victim). So if punishers value longer-than-life sentences, which serve no obvious prospective function, it could be the result not of an explicit, *rational* strategy to communicate, deter, or level the playing field, but of an implicit, *heuristic* one.

Evaluating these various explanations for longer-than-life sentences requires testing an empirical question on which they all rest: do ordinary people, in fact, ascribe value to longer-than-life sentences and treat such sentences as meaningful? Specifically, do they support the use of sentences that far exceed the natural lifespan, and do they adjust these posthumous years in response to mitigating and other contextual factors, as they sometimes do with ordinary sentences? Do they prefer policies that allow life sentences to be ordered consecutively, and are they willing to pay for such policies? Scientists have yet to examine these questions, but their answers could inform the theories, policies, and procedures that dictate how we, as a society, regard and punish violent criminals.

### The Current Study

To test these questions, we conducted two novel survey experiments (see Supplementary Materials for a third experiment [E3] that replicated and extended the second). We favored an experimental methodology because we were interested not only in participants’ conscious attitudes about longer-than-life sentences, but also the underlying causes of their support for such sentences, about which they might not have conscious awareness but can be inferred by random assignment to controlled conditions. All study procedures were approved by the Georgia State University Research Services & Administration office’s institutional review board (#H21668). All measures, manipulations, sample size estimation, and exclusions in the study are disclosed.

In the first experiment (E1), we were interested in how punishment lengths that exceed a human lifespan (the consecutive condition) would compare to those that do not (the concurrent condition). Consecutive and concurrent sentencing practices offer a real-world test of this distinction. We presented laypeople with a hypothetical case summary about an attempted murder with two victims, warranting two sentences in prison that would, if imposed consecutively, exceed the defendant’s life span. Depending on the experimental condition, the instructions explained that those sentences must either be served *concurrently* or *consecutively* (defined as being served “at the same time” or “one after the other”, respectively). Then we asked the participants to make prison sentence-length recommendations, at baseline, and then again after exposure to each of several contextual factors that might provoke a sentencing reduction (i.e., mitigating factors). For those in the *concurrent* sentence condition, their sentencing range options would place the 50-year-old offender between the ages of 60 and 100 at the time of parole, leaving open the possibility that he could be released in his lifetime. For those in the *consecutive* sentence condition, their range of choices was limited to the second sentence only, such that the offender could only be released on parole between the unlikely ages of 110 and 150. The sentencing options in each condition, thus, spanned a 40-year range.

We reasoned that if punishment judgments are based on consequentialist factors, then in serious cases (e.g., with multiple victims), people should not value longer-than-life sentences, since such sentences provide no apparent benefit over the option of a single life sentence. So, given a consecutive life sentence option, they would *not* show a systematic preference for the upper (posthumous) end of the sentencing scale (in the 110-to-150 age range). Instead, they would tend to favor the lower end of the scale, or would choose sentences at random, since they are immaterial.

However, if punishment judgments rely largely on *heuristic* reasoning processes, as we predict, then for serious crimes (e.g., with multiple victims), people should prefer sentences on the upper end of the scale, even when that scale represents years that are posthumous and there is no risk that the offender would be released. Further, these posthumous (consecutive) sentencing recommendations, like their real (concurrent) counterparts, should be responsive to sentence-reducing factors, but only modestly so, signifying that punishers trade off posthumous years *as if* they are material. (For evidence that common “mitigating” factors often have mitigating effects in real cases, see e.g., [29,30]). Sentence-reducing factors should thus exert similar effects under both concurrent and consecutive conditions, in this view. Finally, explicit justifications for heuristic punishments should be retrospective, on the basis of retributive concerns, such as how much the offender deserves the punishment, rather than on the basis of any concrete prospective functions, such as deterrence.

Understanding ordinary people’s sentencing attitudes is important because these attitudes are likely to drive civic engagement, including voting behavior. To test whether sentencing attitudes, like those we describe below from E1, generalize to real-world behavior, we conducted two additional experiments, one using a nationally representative sample of U.S. adults (E2) and the other using an undergraduate sample reported in Section S2 of the Supplementary Materials (E3). In both, participants were asked to express their sentencing attitudes in a more ecologically realistic way, namely, to vote on a hypothetical criminal justice measure. The measure was crafted to *allow* judges to order (or to *prohibit* judges from ordering) *consecutive* life sentences that would extend beyond the lifespan for defendants convicted of first-degree murder and other serious crimes against the same victim (E2) or convicted of the murder of multiple victims (E3). We varied the crime/victim ratio between these voting experiments to explore potential differences in responses that would suggest different psychological mechanisms driving support for longer-than-life sentences. For example, if people only support consecutive life sentences for offenses with multiple victims, this could suggest that each sentence is being used strictly to symbolize each life lost. Within each experiment, we manipulated (1) the presence or absence of implementation costs for the policy change, which is potentially relevant to consequentialist considerations, and (2) the default choice frame (to allow vs. prohibit), a contextual factor that is theoretically irrelevant with respect to consequentialist considerations. E2 predictions and methods were preregistered on Open Science Framework on 8 March 2023 at https://doi.org/10.17605/OSF.IO/U8SAE, accessed on 8 March 2023.

If people support consecutive life sentence policies even when these policies are costly, this would suggest that they find these policies to be valuable. But the expected benefits of adopting such a policy are not easily explained on consequentialist grounds. If their support for these policies persists independently of the default choice (i.e., a contextual frame), we can infer that their choice is not merely a general sensitivity to contextual information (see [31]). (We are not assuming that sensitivity to a heuristic supporting consecutive sentences implies the adoption of heuristics of all kinds. Rather, we assume that different heuristics can compete with each other. The default choice manipulation, thus, allows us to test whether support for consecutive sentencing persists above and beyond other possible heuristic influences.) We predicted that participants would favor the option to allow consecutive life sentences, regardless of the implementation costs or the default choice. Caution is always warranted when interpreting novel lines of research, but if participants across these experiments show a preference for longer-than-life sentences in the ways we predict, it would provide at least suggestive evidence that these punishment attitudes are shaped more by heuristic than consequentialist reasoning.

## 2. Experiment 1

### 2.1. Materials and Methods

#### 2.1.1. Participants

Participants were 187 university undergraduates who received credit for their participation in a course in psychology, philosophy, or political science. Thirty-nine were excluded for incomplete data; four for failing a multiple-choice attention check (“What are the colors of the American flag?”); 10 for failing the catch question instructing to skip the question; two for failing to recognize the correct crime in a multiple-choice list; and two for reporting an age lower than 18. The remaining 130 participants (our final sample) self-identified as 57.7% female, 41.5% male, and 0.8% unanswered; 15.4% Hispanic or Latino; 17.7% White/Caucasian, 40.0% Black or African American, 26.2% Asian, and 6.2% other or unanswered (ethnic and racial categories were non-exclusive); and with a mean age of 20.41 years (*SD* = 5.71).

We are not aware of an a priori power analysis convention specifically for the robust linear mixed-effects model. Hox [32] reports that a rule of thumb for a linear mixed model is at least 10 observations per group. A more conservative approximation might be a linear regression comparing differences between slopes. Under this model, we used G*Power 3 [33] to estimate a minimum sample size based on the smallest effect we were interested in detecting for this first-of-its-kind experiment, which was a minimum slope of 0.1. Our analysis yielded a minimum N = 80 (40 per group), given the following additional assumptions: two-tailed test; alpha: 0.05; power: (1-B) = 0.8; allocation ratio: 1:1; standard deviation of residuals: 3.6; standard deviation for each slope: 23. Post hoc sensitivity analyses were determined to be inappropriate for our experiments (see [34,35,36]).

#### 2.1.2. Design and Procedure

The study utilized an experimental vignette method, delivered online using the *Qualtrics XM* survey platform. Participants read a criminal case summary describing a fictitious offender who qualifies for multiple prison sentences after being convicted of attempting to murder two children. Depending on their randomly assigned condition, participants were informed that, in their jurisdiction, when an offender qualifies for multiple prison sentences, those sentences are treated as either *concurrent* (served at the same time) or *consecutive* (one after the other). The instructions emphasized that the offender either *could* be released on parole in his lifetime (concurrent condition) or could *not* be released in his lifetime (consecutive condition). The dependent measure was the prison sentence recommendation. To make these two conditions commensurable, in the consecutive condition, only the second term was available to be judged. In both conditions, the first 10 years of the prison term was non-negotiable. Thus, the prison sentence measure was presented (on a ratio scale) from 10–50 years in the concurrent condition, or an equal length range of 60–100 years in the consecutive condition. Real jurisdictions, of course, vary in their sentencing ranges for attempted murder. Our primary interest was to compare judgments about concurrent vs. consecutive sentences, so it was important to use sentencing ranges of equal length.

This sentencing measure was administered five times: at baseline and after being presented with each of four contextual factors designed to provoke a sentence reduction, where participants had an opportunity to change their previous sentence or keep it the same. The four sentence-reducing factors were presented in a single fixed order and portrayed evidence of the following: the offender had become rehabilitated and regretted his crimes (“Rehabilitation”); he had suffered a traumatic brain injury that contributed to his crime (“Brain damage”); his sentence was unfair because it was longer than that of even more serious offenders “Unfair”; or his sentence was so long that it would reduce trust in the justice system (“Trust”). The fairness item was included to examine relative comparison to punishment norms (see [37]). The trust item was included to capture theories that punishment doctrines that deviate widely from citizens’ values could reduce trust in and respect for the justice system if viewed as a failure of the system to deliver on its promises [38]. (Technical constraints precluded randomization of the order of presentation of these sentence-reducing factors without violating other survey features (i.e., response piping). Lacking strong theoretical interest in the independent effects of each reduction type, we chose a fixed order to ensure that participants could receive a visual reminder of their previous sentence recommendation. Results should be interpreted with this limitation in mind.) Our experiment thus followed a 2 between-subjects sentence type [Concurrent (N = 64) vs. Consecutive (N = 66)] × 5 within-subject reduction context [Baseline, Rehabilitation, Brain damage, Unfair, Trust] mixed design. (See Section S1 for exact stimuli).

Following the sentencing measures, participants answered several questions. The first asked them to describe why they chose the sentence that they recommended (free response). We used these responses to examine how participants’ justified their punishments and whether those justifications varied as a function of our concurrent vs. consecutive sentencing conditions. Their punishment justifications were coded by two trained raters (all *k* ≥ 0.80), blind to our study hypotheses. The raters coded their responses according to the presence (‘1’) or absence (‘0’) of the following non-mutually exclusive categories: retributive/deontological (deserts, proportionality, moral or legal obligation), consequentialist (incapacitation, deterrence, rehabilitation), communicative (to express condemnation to society), comments that the specific sentence was arbitrary (e.g., because the offender will not live that long, anyway), unjustified assertions, and negative justifications (i.e., reasons to support a sentence reduction). These categories were defined on the basis of theoretical factors and a preliminary review of a subset of the responses. Notably, retributive sentiments and unjustified assertions would be interpreted as most consistent with the heuristic processing perspective (see [21]). The two raters’ dichotomous scores for each category were summed into a single composite variable (0–2) from which frequencies and mean scores were computed.

The next set of questions assessed agreement with the assumptions of our manipulations, allowing us to test these assumptions (e.g., “Judges should be allowed to impose prison sentences that exceed the human lifespan”, “Mr. Smith deeply regrets the harm he caused”) on a 7-point ordinal scale from “strongly disagree” (−3) to “strongly agree” (+3).

Participants then indicated their general support for common philosophical justifications for punishment, namely, retributive (+1) vs. consequentialist (−1), using a structured scale adapted from [39], and indicated their degree of support for that choice on a 5-point scale from “not strongly at all” (1) to “very strongly” (5). Retributive purpose: “...to give them what they deserve, to condemn them for what they’ve done, and to restore moral order in the community”; consequentialist purpose: “...to discourage them from committing more crimes, to discourage others from committing crimes, and to rehabilitate them”; or “None of the above statements represent my views on punishment”. A summary score was generated by computing the product of the selected philosophical justification by the degree of support. This score was used to test potential associations with their specific sentencing recommendations.

For exploratory purposes, participants completed a 15-item scale assessing beliefs about free will, determinism, and dualism using the Free Will Inventory (e.g., “People always have the ability to do otherwise”; [40]). Only the “free will” subscale was analyzed (items 1, 4, 7, 10, and 13). Mean scores for this subscale were calculated to explore possible associations between the belief in free will and support for consecutive life sentences, since previous studies, grounded in desert-based theories, suggest that stronger belief in free will may be associated with increased punishment judgments, under some conditions (e.g., [41,42,43]).

Last, standard demographic information was assessed (gender, age, race, political ideology, and political party). (Two additional scales—a measure of intellectual humility [44] and an internally developed measure of implicit punishment motives—were included after the manipulation checks for an unrelated study and were not included in the present analysis.) The original data and supporting materials for this study are openly available on Open Science Framework at http://doi.org/10.17605/OSF.IO/YKMNV (accessed on 21 August 2024).

### 2.2. Results

#### 2.2.1. Punishment Behavior

Our primary aim was to explore whether people who are limited to a posthumous sentencing range (the consecutive group) will punish in a manner similar to those limited to a range of real-life years (the concurrent group), consistent with the heuristic processing perspective. Specifically, at baseline, we predicted that each group will punish at levels substantially greater than the scale minimum, and they will reduce their sentences in response to our contextual factors (i.e., a negative effect) but only to an intermediate degree (i.e., not as far as the scale minimum, paralleling the partial sentencing reductions so common among ordinary, less-than-life sentences with compelling mitigating factors).

To test these predictions, a robust linear mixed-effects model was constructed using the robustlmm, lme4, and lmerTest packages [45] in R v. 4.2.2 [46]. The model assessed the association between sentence type (Consecutive or Concurrent) and reduction context (Baseline, Rehabilitation, Brain damage, Unfair, or Trust) as fixed effects, and sentence length (measured in years). Participant identifier was entered as a random effect to account for different baseline rates in sentencing (See Table 1). Robust linear mixed-effects models are an extension of standard linear mixed-effects models that are less sensitive to outliers and other data issues that may bias model parameter estimates [47]. These models also overcome rigid assumptions of linearity and independence inherent to traditional Analysis of Variance methods [48,49,50]. Reduction context was modeled with polynomials up to fourth-order effects. An additional set of interaction terms modeled the difference across reduction contexts between sentence types. All post hoc analyses were conducted with a false-discovery rate (FDR) correction.

**Figure 1:**
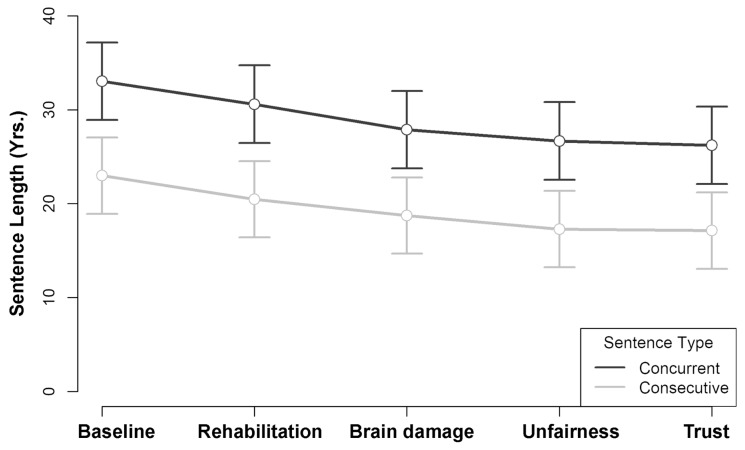
Sentence length recommendations (in years) at baseline and after exposure to each of four reduction contexts for participants provided with a concurrent sentencing range (black) and a consecutive sentencing range (gray). Each sentencing scale is yoked to the scale minimum, here denoted as zero. Error bars represent 95% CI.

Figure 1 shows the estimated association between sentence length recommendations across reduction context by sentence type. Because the absolute values differed by condition, the figure illustrates their relative differences only by adjusting each scale to zero. On average, participants assigned to the concurrent condition punished in greater proportion (relative to the scale minimum) than those assigned to the consecutive condition (estimate = 9.55 yrs., *z* = 3.30, *p* = 0.001). This initial finding is not surprising because the former were limited to a much lower maximum scale value (50 yrs.) than the latter (100 yrs.).

In testing the polynomial relationship between sentence length and sentence type for concurrent sentences, we found a significant negative linear effect (estimate = −5.55 yrs., *p* < 0.001) and positive quadratic effect (estimate = 1.45 yrs., *p* = 0.002) (See Table 1). This pattern was replicated in consecutive sentences. As seen in Figure 1, this pattern of significance indicates a leveling-off relationship with additional sentence-reduction contexts. Across conditions, the average baseline sentence (*M* = 28.0 yrs., 95% CI [25.1, 30.9]) was associated with longer sentences than Rehabilitation (*M* = 25.5 yrs., 95% CI [22.6, 28.4], *z* = 5.46, *p* < 0.001), Brain damage (*M* =23.3 yrs., 95% CI [20.4, 26.2], *z* = 10.35, *p* < 0.001), Unfairness (*M* = 22.0 yrs., 95% CI [19.1, 24.9], *z* = 13.23, *p* < 0.001), and Trust (*M* = 21.7 yrs., 95% CI [18.8, 24.6], *z* = 13.97, *p* < 0.001).

Comparisons between reduction contexts should be interpreted with caution, since our survey was limited to a single fixed order. The first reduction context (Rehabilitation) was associated with longer sentences than the second (Brain damage: *z* = 4.89, *p* < 0.001), third (Unfairness: *z* = 7.80, *p* < 0.001), and fourth (Trust: *z* = 8.51, *p* < 0.001); and the second (Brain damage) was associated with longer sentences than the third (Unfairness: *z* = 2.91, *p* = 0.004) and fourth (Trust: *z* = 3.62, *p* < 0.001). These effects could either be due to the influence of the factor itself or to a property of the order in which it appeared. A significant difference was not found between the last two mitigation types (Unfairness and Trust: *z* = 0.71, *p* = 0.477).

The more relevant comparison in this analysis is between these reduction types as a whole and the baseline sentence. For both concurrent (−6.82 yrs., *p* < 0.001) and consecutive sentences (−5.85 yrs., *p* < 0.001), there was a significant amount of total change from Baseline to Trust sentences. Importantly, the lowest mean scores (following the Trust factor) were substantially higher than the minimum scale value, both for those judging concurrent life years, *t*(63) = 14.42, *p* < 0.001, *MD* = 24.93 yrs., 95% CI [21.47, 28.38] and those judging consecutive life years, *t*(65) = 8.24, *p* < 0.001, *MD* = 16.12, 95% CI [12.21, 20.02]. In other words, both groups exhibited high amounts of punishment initially, and as predicted, were partially, but not drastically, responsive to sentencing reduction contexts, with average sentences in the consecutive condition never dropping below a sentence that would be completed before the offender would reach 115 years old.

The finding that sentencing patterns in the consecutive condition mirrored those in the concurrent condition is consistent with the operation of a decision-making heuristic that generalizes punishment preferences from a real-world domain to a symbolic one.

To explore other potential evidence of heuristic processing, we examined patterns of clustering and dispersion in our sentencing recommendations. Previous research has found that judicial sentencing decisions tend to cluster around whole numbers and even numbers, even though there is no formal legal reason for doing so (e.g., [26]). This evidence that judges prefer round numbers has been interpreted as evidence of heuristic processing [51]. To assess the presence of rounding in our sentencing recommendations, we conducted a Two-Step Cluster Analysis. Across conditions, our results show a robust pattern of rounding to the nearest increment of five, consistent with the heuristic processing perspective. (See Section S2 for analysis).

Individual difference variables were assessed for associations with our baseline sentencing measures using separate Pearson correlation analyses. Consecutive, longer-than-life sentencing recommendations were positively associated with the male gender, *r*(64) = 0.28, *p* = 0.022, beliefs in free will, *r*(63) = 0.26, *p* = 0.039, and a more conservative political ideology, *r*(53) = 0.38, *p* = 0.005. They were not associated with age, *r*(46) = −0.22, *p* = 0.126. Prison sentences in the concurrent condition were not associated with age, *r*(41) = −0.03, *p* = 0.839, gender, *r*(62) = −0.05, *p* = 0.680, or beliefs in free will, *r*(61) = 0.06, *p* = 0.619, and were positively associated with a more liberal political ideology, *r*(47) = −0.29, *p* = 0.045.

#### 2.2.2. Manipulation Checks

The results of our hypothesis test comport with the results of our manipulation check questions, as demonstrated by a set of one-tailed, one-sample *t*-tests (*df* = 129) to assess explicit agreement with the arguments for a sentencing reduction (See Table 2). Consistent with their sentencing reductions following the Rehabilitation factor, participants agreed with the statement that the defendant deeply regrets the harm he caused; they agreed that he has difficulty controlling his impulses, consistent with their sentence reductions following the Brain damage factor; they agreed that it would be unfair if others received lighter sentences for more harmful crimes, consistent with their sentence reductions following the Unfair factor; and they neither agreed nor disagreed with the statement that lengthy sentences could reduce the public’s trust in the justice system if those sentences often exceed a defendant’s life expectancy, which is consistent with the null effect of the Trust factor on their sentences. These reported beliefs did not differ by experimental condition (Table 2). This pattern implies that, with the exception of the Trust factor, the reduction context information worked as intended, and that participants’ beliefs in these propositions can help to explain the sentencing reductions observed across conditions.

Participants’ normative attitudes also aligned with their sentencing behavior in a manner consistent with the heuristic processing perspective. As a whole, they expressed agreement with statements that judges should be allowed to impose consecutive life sentences, but not necessarily that judges should be allowed to impose concurrent life sentences (see Table 2). This pattern, which mirrors their sentencing behavior, implies that they recognized the difference between these two sentencing types and favor the option of consecutive life sentences even when a concurrent life sentence would have been sufficient to incarcerate the offender to the age of 100.

On average, they did not agree with the statement that “I would be satisfied if [the defendant] was given only one 50-year prison sentence” (See Table 2). Interestingly, their answers to this question varied as a function of the sentence type, such that those in the concurrent condition agreed (*M* = 0.64, *SD* = 1.85), but those in the consecutive condition did not agree (*M* = −0.83, *SD* = 1.78). This pattern implies that having access to the option of a consecutive life sentence itself might reduce satisfaction with a *concurrent* sentencing policy. One way to interpret this finding, if we take their responses at face value, is that people given a consecutive sentencing policy are poor at predicting how they would feel about concurrent life sentences: they expect they will be dissatisfied, but when placed under that constraint they are not explicitly dissatisfied, on average.

#### 2.2.3. Follow-Up Questions

To investigate why participants might favor longer-than-life sentences, we analyzed participants’ written justifications for their specific punishment recommendation, comparing frequencies of different justification types: retributive (e.g., “because he deserved it”), consequentialist (e.g., “because he could do it again”), communicative (e.g., “to illustrate the severity of his crimes”), arbitrariness (“because it doesn’t matter what you choose, he’ll more than likely die before the sentence is up”), unjustified assertions (e.g., “Why not” give a harsh sentence), and negative justifications, namely, reasons to support a sentence reduction (e.g., “The focus should be on rehabilitation”). Collapsing across our experimental conditions, we found that retributive justifications (*M* = 1.08, *SD* = 1.00) were far more frequent than all other justification types. Communication (*M* = 0.04, *SD* = 0.26) was the least frequent, and the other types of justification did not statistically differ from each other in frequency: consequentialist (*M* = 0.25, *SD* = 0.66); arbitrary (*M* = 0.26, *SD* = 0.68); unjustified assertion (*M* = 0.41, *SD* = 0.80); and negative reason (*M* = 0.18, *SD* = 0.57) (see Table 3 and see Table S1 in Section S2).

When comparing experimental conditions, the retributive justification was disproportionately represented in the concurrent sentencing condition compared to the consecutive condition, *t*(128) = 2.33, *p* = 0.021, *d* = 0.41. None of the other comparisons were statistically significant: consequentialist, *t*(128) = −1.39, *p* = 0.166; communicative *t*(128) = −1.66, *p* = 0.099; unjustified assertion, *t*(128) = −0.67, *p* = 0.502; negative justification, *t*(128) = −0.25, *p* = 0.802. The apparent decrease in retributive sentiments in the consecutive condition could be explained by an increase in comments that the sentence chosen is arbitrary, *t*(128) = −2.30, *p* < 0.023, *d* = −0.40, with several participants explicitly arguing that the specific sentence does not matter because the offender will not live that long (e.g., “If he is expected to die in this time frame, there is no reason to extend.”). Despite the increase in arguments about the arbitrariness of the punishment choice, retribution still prevailed as the leading justification in each condition, concurrent and consecutive. (See Table S1 in Section S2).

Our core finding in Experiment 1 was that, at baseline, participants who were given a consecutive sentencing option, much like their counterparts in the concurrent condition, demonstrated a preference for sentences in the upper end of the sentencing scale, even though such a preference serves no obvious consequentialist function. To the contrary, participants’ justifications for these preferences were distinctly retributive. Moreover, when given an opportunity to consider mitigating circumstances, they reduced their sentencing time recommendations at a rate that was indistinguishable from the mitigation rate observed by those in the concurrent condition. This pattern cannot be easily explained by consequentialist theory. Instead, it suggests that participants traded off these longer-than-life years as if they were material, as we would expect of a (retributive) heuristic that extends a material decision-making process to the symbolic domain.

## 3. Experiment 2

The purpose of E2 was to test laypeople’s support for consecutive life sentencing in a more ecologically relevant task: voting decisions. A nationally representative sample of adults voted on a hypothetical criminal justice measure about whether to allow judges to order consecutive life sentences, as opposed to concurrent life sentences, for defendants convicted of multiple serious crimes against the same victim. (Compared to E3, which presented a similar hypothetical sentencing measure but involving serious crimes against multiple victims, permitting us to explore the specificity or generality of support for consecutive life sentences.) Because it is possible that other factors, such as the implementation costs of the proposed policy (a factor that is potentially relevant to consequentialist considerations) and whether the policy is presented as the default or the alternative choice (a contextual frame that is irrelevant to consequentialist considerations), could exert independent effects on support for consecutive life sentences, we tested these influences as well. We predicted in a preregistration that participants would favor the option to allow consecutive life sentences, regardless of whether implementing the policy was costly to taxpayers or whether it was the default choice frame. Such a finding would support the notion that punishment attitudes are shaped by a heuristic reasoning process specifically related to retributive motives rather than consequentialist motives or irrelevant contextual framing.

### 3.1. Materials and Methods

#### 3.1.1. Participants

Participants were 234 adults recruited on *CloudResearch Connect* and sampled to be representative of the U.S. population for the attributes of age, sex, and race. In accordance with our planned exclusion criteria, 3 were excluded for incomplete data; 33 for failing two multiple-choice manipulation checks; 11 for failing to recognize the correct definitions of “consecutive” and “concurrent” (i.e., life sentences that are served “one after another” or “at the same time”); and two were excluded for failing a multiple-choice attention check (same as E1). The remaining 182 participants (our final sample) reportedly were 49.2% female, 48.8% male, and 0.8% other or unanswered; 15.1% Hispanic or Latino; 73.4% White/Caucasian, 14.7% Black or African American, 4.4% Asian, and 2.4% other or unanswered (ethnic and racial categories were non-exclusive); and with a mean age of 46.9 years (*SD* = 16.1).

A minimum sample size estimate was based on an a priori power analysis, using a Chi-square test of independence with one degree of freedom to compare frequencies of vote types as a function of our two manipulations, assuming a medium effect size (w = 0.30), which was derived from internal pilot testing. Assuming an alpha threshold of 0.05, 145 participants would be sufficient to detect an effect at a power threshold of 0.95. We sought to exceed this number, to account for possible attrition and data exclusion.

#### 3.1.2. Design and Procedure

As in E1, participants were surveyed online. After brief initial instructions, they were asked to read a fictitious ballot measure either to allow or to prohibit (as determined by random assignment) consecutive life sentences for felons convicted of multiple serious crimes against the same person. Within each of these conditions, half the participants were told that the new policy would cost $10 million USD in judicial training costs, to be paid by taxpayers in their state. The other half were told that the new policy would place no financial burden on the state or taxpayers. This structure comprised a 2 (allow vs. prohibit) × 2 (cost vs. no cost) between-subjects factorial design.

The dependent measure was a dichotomous “yes/no” choice common among many real ballot measures (“Shall the State adopt this measure?”). The default choice was counterbalanced for control purposes. In the allow condition, a “yes” vote would allow judges to begin ordering consecutive life sentences in addition to concurrent life sentences, whereas a “no” vote would continue to prohibit them from doing so, allowing concurrent life sentences only (i.e., the default choice). In the prohibit condition, a “yes” vote would prohibit judges from ordering consecutive life sentences, whereas a “no” vote would allow judges to continue to order consecutive life sentences in addition to concurrent life sentences (i.e., the default choice). For exploratory purposes, participants were also asked to rate their degree of support on a 7-point scale (*1* = Strongly Oppose to *7* = Strongly Support). (See Sections S1 and S2 for stimuli and supplementary analyses).

Following the voting measure, participants were asked whether or not certain mitigating factors (such as brain damage) should be considered in judges’ sentencing decisions including consecutive life sentences and parole decisions (yes/no). We reasoned that participants who support consecutive life sentences would be less accepting of a sentence reduction than those who oppose the consecutive life sentence policy, even when restricted to the posthumous range.

Next, participants were asked, on a 7-point scale (*1* = Strongly disagree to *7* = Strongly agree), the extent to which they (dis)agree with the following: offenders given life without parole are unlikely to be released in their lifetime; they would feel satisfied if serious offenders with multiple victims were given only one life sentence without parole; extremely long sentences could reduce the public’s trust in the justice system if these sentences are usually longer than the actual prison time served; and people who commit serious crimes will be punished in the afterlife. These questions were designed to test the assumptions of our manipulations and probe for explanations for our effects.

Using a ranking question, we assessed participants’ attitudes about specific communicative purposes of consecutive life sentences. Then we assessed their explicit punishment justifications by asking them to rank the relative importance of retribution (“we give them what they deserve”), deterrence (“we protect society from future harm”), rehabilitation (“we reform and rehabilitate them”), and communicative justifications (“we send a message that what they did is wrong”) for criminal punishment, in response to the prompt “People who commit crimes should be punished because by punishing them…”, where *1* = ‘agree with most’ and *4* = ‘agree with least’.

Last, we collected demographic information as described in E1 and self-reported socio-economic status (lower, lower-middle, upper-middle, and upper).

### 3.2. Results

#### 3.2.1. Baseline Voting Behavior

First, we sought to determine whether participants would show a preference for the consecutive life sentencing policy over the concurrent one even though the instructions plainly stated that offenders convicted of multiple serious crimes against a victim would never be eligible for parole under either policy. To test the proportion of participants voting to allow consecutive life sentences versus voting to prohibit them, we conducted a Chi-square test collapsing across the default choice frame. Similar to E1, participants exhibited a strong preference for the choice to allow consecutive life sentences, with 73% voting to allow and just 27% voting to prohibit, χ^2^(1, 182) = 38.77, *p* < 0.001, comprising a medium effect size, *w* = 0.46.

#### 3.2.2. Effect of Choice Frame and Implementation Cost

**Figure 2:**
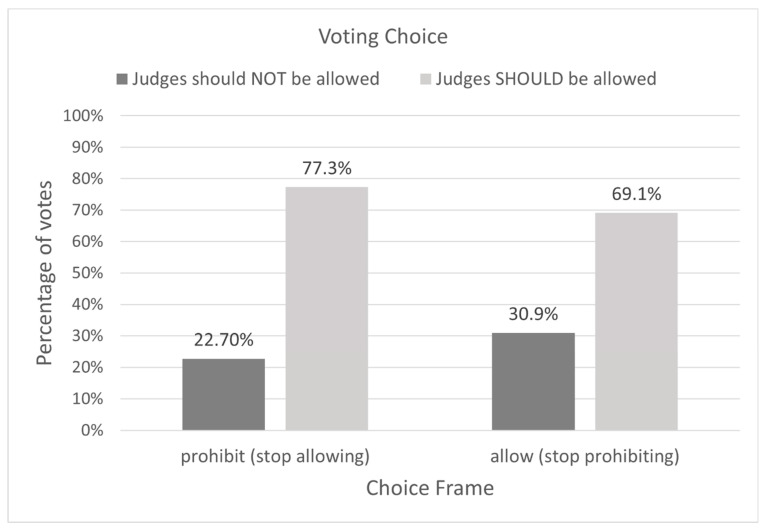
Percentage of participants in each condition who voted for or against the consecutive life sentence policy, showing disproportionate preference for consecutive sentences, regardless of how the choice was framed.

**Figure 3:**
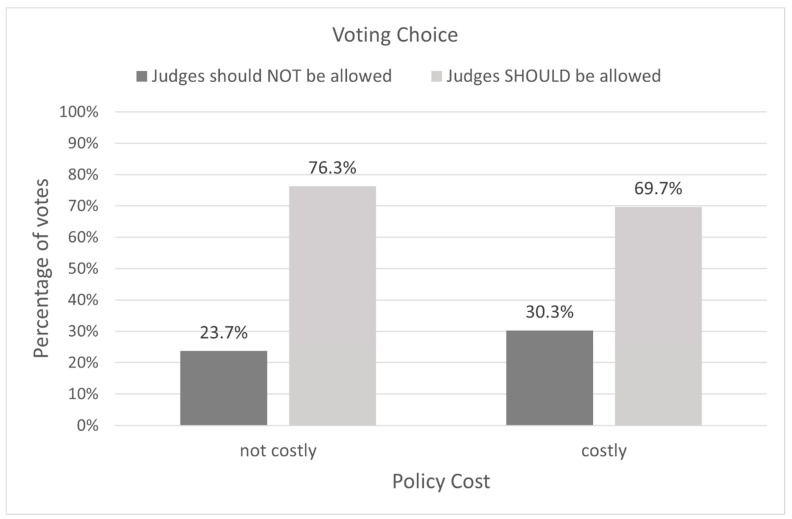
Percentage of participants in each condition who voted for or against the consecutive life sentence policy, showing disproportionate preference for consecutive sentences, regardless of implementation costs.

Next, we examined whether support for the consecutive life sentencing policy depended on our manipulated variables: the default choice and policy implementation costs. Using a Chi-square test of independence, an effect of the default choice frame on voting preference was not found, χ^2^(1, 182) = 1.53, *p* = 0.22. That is, whether the choice was framed as a decision to begin to allow consecutive life sentences or to stop prohibiting them, the preference for the consecutive life sentencing option persisted. Likewise, we found no effect of implementation cost on voting preference, indicating that participants were supportive of consecutive life sentences, regardless of whether the stipulated cost to implement this policy was low or high, χ^2^(1, 182) = 1.03, *p* = 0.31 (See Figure 2 and Figure 3). These findings suggest that our participants valued the consecutive life sentencing option affirmatively, not just by default or when it is cost-free.

#### 3.2.3. Follow-Up Questions

The results of our follow-up questions helped to confirm and explain the observed support for longer-than-life sentences. First, participants as a whole agreed that when an individual commits multiple serious crimes against the same person, it is important to arrange his sentences consecutively in order to do justice for each crime he committed (*M* = 0.71, *SD* = 1.60), *t*(181) = 5.98, *p* < 0.001, *d* = 0.44. Importantly, they also agreed that an offender given a single life sentence is extremely unlikely to get out of prison in his lifetime (*M* = 1.54, *SD* = 1.38), *t*(181) = 15.13, *p* < 0.001, *d* = 1.12, minimizing the concern that their support for longer-than-life sentences was motivated primarily by an incapacitative concern to prevent early release. In addition, supporters of consecutive life sentences were reportedly less satisfied (*M* = 0.20, *SE* = 0.14, 95% CI [−0.07, 0.47]) than opponents (*M* = 1.78, *SE* = 0.23, 95% CI [1.33, 2.22]) by the prospect of serious offenders with multiple crimes against the same victim being given only one life sentence without parole, *F*(1, 180) = 35.73, *p* < 0.001, η_p_^2^ = 0.17.

To explore potential motivations for their sentencing preferences, we assessed agreement with different types of communicative motivations for longer-than-life sentences. Descriptively, the highest-ranked communicative motivation—ranked highest by 32.2% of participants—was that consecutive life sentences, compared to a single life sentence, uniquely communicate to the victim’s family that their loss will be remembered. (Communicating to the offender was ranked highest by 24.9%; communicating to would-be offenders was ranked highest by 22.0%; and communicating to the general community was ranked highest by 7.3% of participants). Perhaps more revealing, a correlational analysis indicated that greater support for the policy was associated with stronger beliefs that these sentences are more effective at communicating to would-be offenders not to commit such crimes, Spearman’s ρ = −0.25, *p* < 0.001. Support was not associated with communicating to the victim’s family, ρ = −0.06, *p* = 0.40, or communicating the wrongfulness of the crime to the offender, ρ = 0.00, *p* = 0.98, or to the general community, ρ = −0.12, *p* = 0.12. Policy support was also associated with the answer option “None of these”, Spearman’s ρ = −0.36, *p* < 0.001, suggesting that participants may have had still other reasons for valuing consecutive life sentences that were not adequately captured by our other communicative answer options, such as, perhaps, notions of retributive proportionality.

Opponents of the consecutive life sentencing policy endorsed different motivations than supporters. Specifically, opponents were more likely than supporters (*M* = −0.64, *SE* = 0.14, 95% CI [−0.92, −0.36]), to agree that prison sentences that are longer than the offender’s life are pointless because they are no more effective than a single life sentence, *F*(1, 180) = 69.14, *p* < 0.001, η_p_^2^ = 0.28 (*M* = 1.65, *SE* = 0.24, 95% CI [1.19, 2.12]). Compared to supporters (*M* = −0.89, *SE* = 0.14, 95% CI [−1.15, −0.62]), opponents were also more likely to agree that imposing extremely long sentences could reduce the public’s trust in the justice system if these sentences are usually longer than the actual time served, *F*(1, 180) = 19.51, *p* < 0.001, η_p_^2^ = 0.10 (*M* = 0.27, *SE* = 0.22, 95% CI [−0.18, 0.71]), pointing to a potential affirmative motivation for their opposition. The two groups did not statistically differ in the belief that people who commit serious crimes will be punished in the afterlife, *F*(1, 180) = 0.03, *p* = 0.88 (*M* = 0.05, *SD* = 1.80), weakening the conjecture that such beliefs could decrease their support for longer-than-life sentences.

As a whole, participants were relatively willing to consider a sentence reduction in light of mitigating circumstances (such as brain damage), but only within the posthumous range, χ^2^(1, 182) = 1.41, *p* = 0.24, rather than extending into the range of real-life years, χ^2^(1, 182) = 16.02, *p* < 0.001. When considering mitigation within the posthumous range, those who supported consecutive life sentences were less accepting of a sentence reduction (48.1% accepted) than those who opposed the policy (71.4% accepted), χ^2^(1, 182) = 7.84, *p* = 0.005. This pattern suggests that participants still found the practice of mitigation to be meaningful when it was restricted to being within the posthumous range.

Support for the consecutive life sentencing policy was not associated with age, *r*(137) = 0.00, *p* = 0.997, gender, *r*(180) = −0.083, *p* = 0.266, political ideology, *r*(178) = 0.141, *p* = 0.059, or socio-economic status, *r*(180) = −0.016, *p* = 0.835.

## 4. Discussion

### 4.1. Conclusions

The purpose of this study was to examine whether most people favor the use of longer-than-life sentences, and why. From a strict consequentialist perspective, these longer-than-life sentences carry no more value than a single life-long sentence without the possibility of parole. But if people demonstrate systematic preferences for longer-than-life sentences and apply them in a similar way as real sentences, then this would imply that they ascribe value to the posthumous portion of such sentences and are reasoning about them using the same decision rules they use for less-than-life sentences. Such a finding is difficult to square with consequentialist accounts of our punishment psychology.

On the whole, our results appear to be more consistent with the heuristic processing perspective (see also [8,51]). As predicted by this perspective, participants in all the experiments showed a preference for longer-than-life sentences even when there was no possibility of parole. In E1, participants in both conditions (concurrent and consecutive) favored the upper end of the sentence scale, that is, regardless of whether it represented life years that were actual and could be served or posthumous years that could not be served. The two sentencing functions, across reduction contexts, had very similar slopes, which did not statistically differ from each other. Both slopes best fit a negative linear function but can also be described as curvilinear, leveling out well above the scale minimum. As such, both groups were partially receptive to sentence-reducing contextual factors, but the force of these factors dissipated as the number of contextual factors increased. This reduction in sensitivity to contextual factors suggests a pattern of trading off with posthumous life years that resembles real-life-year mitigation in sentencing decisions—except without any obvious practical application.

There was a main effect of sentence type in E1, where punishments in the concurrent condition were closer to the ceiling than those in the consecutive condition, but this is not surprising because the anchor points on the concurrent scale were much lower. One obvious explanation for this pattern could be error management—a strategy for reducing the real possibility of a premature release in that condition. But this pattern in no way undermines the heuristic processing theory, which made no predictions about the relative positions of the sentencing means across the two scales. Rather, that theory is concerned only with the absolute positions on each scale, as well as the shape of each curve. These data strongly suggest that our participants engaged in tradeoffs with posthumous life years similarly to that of real-life years. Consequentialist theories do not provide a direct way to explain such a pattern.

E2 reproduced this basic pattern in a voting context involving life sentences without the possibility of parole in a nationally representative U.S. sample. We found that participants exhibited a strong preference for a policy that would allow (or stop prohibiting) consecutive life sentences for eligible offenders. Their support persisted even when the policy was portrayed as costly to implement and the default choice varied, consistent with other punishment research (e.g., [52,53]).

The results of E2 were successfully replicated in E3, using crimes against multiple victims rather than multiple crimes against a single victim (see Section S2). The fact that participants in these experiments were willing to incur a cost to enact the policy and to do so under different choice frames implies that their support was not arbitrary or made by default, but instead was genuine and robust. Furthermore, their support was not merely a tactic to hedge against a faulty justice system that might release dangerous criminals from their sentences because these participants showed strong agreement with the assertion that offenders who are sentenced to life without parole are extremely unlikely to get out of prison in their lifetime. The fact that they supported posthumous sentences under these conditions and without any obvious practical reason is more consistent with the operation of a heuristic reasoning process. One might wonder why concurrent life sentences of real years cannot have the same symbolic value as consecutive life sentences. Heuristics may not be responsive to such considerations.

Participants’ expressed preferences generally cohered with their punishment behavior. In E1, when they explicitly agreed with a sentence-reducing factor, they likewise reduced their punishment recommendation, and when they did not accept a sentence-reducing factor, they recommended lengthy punishment. The fact that this pattern extended to the consecutive condition, when doing so was materially inconsequential, implies that they might not have experienced significant decision conflict about the justification for these posthumous sentences. They indicated, in all experiments, that judges should be allowed to impose longer-than-life sentences. In other words, they endorsed longer-than-life sentences even though such sentences are not justified by consequentialist reasons, consistent with a heuristic punishment process.

Analysis of participants’ written justifications aligns with this interpretation. For example, participants most frequently cited *retributive* reasons for their specific sentencing recommendations (E1), consistent with previous research (e.g., [10,21,54]). Those who supported the consecutive life sentencing policy were more likely to endorse communicative justifications for such sentences (E2 and E3), but evidence for consequentialist justifications was weak or mixed (see Section S2 for analyses). Each experiment also revealed a modest increase in other justification types. For example, in E1, the second most prominent justification for punishment consisted of unjustified assertions (e.g., “Why not” give a harsh sentence). This implies a potential lack of awareness of one’s reasons for supporting posthumous punishment, consistent with previous research on retributive punishment [21]. E1 also revealed in the consecutive condition a relative increase in comments that the specific sentence length does not matter (i.e., is arbitrary) because the offender will not live long enough to serve it. These attitudes can be interpreted as consequentialist because they assume that sentences should have a forward-looking, practical function. Yet, despite this relative increase in consequentialist justifications, non-consequentialist justifications were more prominent, implying that most participants justified their punishments as if they believed that posthumous sentences can be more effective than a true life sentence at denouncing the crime and restoring justice.

In E2 and E3, the goal of “sending a message” was disproportionately endorsed by supporters of the consecutive life sentencing policy, suggesting that the communicative function of punishment may play a role, along with retribution, in support for consecutive life sentences. This pattern is consistent with other research findings that Americans endorse a variety of punishment motives, including communicative ones, at least when assessed using self-report (e.g., [55]). Pluralistic punishment motivations could also explain why we did not observe a punishment justification preference using the forced-choice justification question. Perhaps, in this explicit format, it was hard for people to choose. E2 also helped us address the question of what it means to participants to “send a message” (e.g., to whom and why), showing that support for consecutive sentences was positively associated with the belief that they are more effective at communicating to would-be offenders not to commit such crimes. From a strict consequentialist perspective, it remains unclear why people think these sentences are more effective deterrents, given that no offender could ever complete more than one life sentence. Still, it is clear that our participants preferred longer-than-life sentences, and they did so for reasons that appear to be more symbolic than instrumental.

This study also enabled us to assess whether support for consecutive life sentences is uniquely calibrated to represent the lost lives of each victim (as in E3), or whether it also responds to other types of violations, in this case, multiple serious crimes against a single victim (E2). We found that support did, in fact, track multiple crimes against the same victim, suggesting that multiple goal representations can trigger support for longer-than-life sentences, perhaps as part of an implicit assessment of the combined moral gravity of the offenses or the representation of the overall harm caused.

In E1, sentencing recommendations in the consecutive condition were positively associated with self-reported political conservatism. This is consistent with characterizations of conservatives as more retributive than liberals (e.g., [56]). In E1 and E3, free-will beliefs were positively correlated with support for consecutive life sentences. This correlation is consistent with previous studies of less-than-life punishments, in which increased belief in free will correlates with increased punishment (e.g., [41,42,43]). This finding accords with desert-based theories that acting on free will is a precondition for blame and punishment—but, given the correlational nature of our analysis, other explanations are possible, such as that the attribution of free will was constructed to satisfy a pre-existing desire to blame and punish (see [41,57]). Our exploratory analysis cannot distinguish between these explanations.

### 4.2. Future Directions

This project and its measures sometimes assumed clear distinctions among consequentialist, retributive, and communicative punishment theories and punitive motives. However, we take this assumption to be provisional, because the traditional legal punishment framework could be flawed (see [20]). For example, the same punishment can communicate both messages of deterrence and moral condemnation, and retributive judgments can have consequential effects on the offender’s future behavior. (For examples of differing characterizations of the expressive functions of punishment, see [58,59]). Similarly, we argued that retributive and communicative theories might operate more heuristically in certain cases where the direct justification for such punishments is hard to find. Yet, consequentialists can plausibly make heuristic judgments, too [60,61]. Our findings do not refute these challenges, but to the extent that punishments do vary in their motivations and in their degree of conscious reflection, our findings suggest that the special case of longer-than-life sentences could potentially be used as a way to discern such variation (see [62], for an analogous argument based on punishment attitudes toward non-human animals). Toward this end, much research, both conceptual and empirical, will be needed. We outline some practical recommendations below.

In order to draw causal inferences about the effect of a potential sentencing policy on punishment behavior, this experimental study was restricted to the measurement of hypothetical judgments by layperson samples, which may or may not generalize to real-world sentencing attitudes. Future research can overcome these limitations by surveying professional judges or by analyzing real-world legal data, perhaps by comparing sentencing determinations between jurisdictions that allow vs. prohibit consecutive life sentences for capital crimes or longer-than-life sentences for violent but non-capital offenses. Although such techniques preclude causal inference, convergent results between these separate methods would serve to validate our conclusions. Until then, our findings serve as a proof of concept that the systematic measurement of attitudes toward longer-than-life sentences can shed light on the extent of support for such sentences and reasons for supporting them.

In E1, we found, as predicted, that the observed negative slope in sentencing appeared in both concurrent and consecutive conditions. It is still possible that there were differences between the curves that we did not detect. Testing for all such possible differences was beyond the scope of our theoretical interests for this experiment. However, future investigations should consider the more ambitious, affirmative test of the null hypothesis that there are no differences between concurrent and consecutive sentencing behavior. We regard such a conclusion as a stronger claim than we make here, namely, that some meaningful properties of consecutive life sentencing behavior appear to replicate those of concurrent life sentencing behavior.

In the same experiment, because the first sentence in the consecutive condition was mandatory, it could have anchored participants’ sentencing judgments for the second sentence by suggesting what constitutes a “fair” punishment for the first victim. This could potentially explain why participants in the consecutive condition engaged in posthumous punishment (see [22,63]). This explanation is unable to explain the mitigation pattern we observed across conditions or the retributive motivations expressed in participants’ self-reported justifications. It also cannot explain the strong support for the consecutive sentencing policy observed in E2. However, if participants were anchored in this way, it could provide another heuristic-based explanation for the baseline punishment behavior. Future research could attempt to control for this potential confound by equating the baseline sentences rather than the length ranges.

Sentencing defaults vary by state and by the elements of the crime. Thus, a secondary question addressed by E2 and E3 was whether support for consecutive life sentences depends on the default choice frame: whether the dichotomous choice is framed as a decision to switch from allowing to prohibiting such sentences, or vice versa. Although we did not find direct evidence that the framing type moderated voting choices, exploratory analyses using a continuous measure of support were mixed (see Section S2). If such effects exist, they would have direct relevance to sentencing policy design and communication. Future research on life sentencing attitudes, therefore, should more rigorously test the potential moderating effects of choice frames. In a similar vein, our tests of single-victim and multiple-victim scenarios yielded comparable effects, but interpretation might depend on the way these scenarios were presented (i.e., separately). Other research suggests that separate and joint presentation formats have distinct effects on willingness to help victims [64]. By extension, joint evaluation of the number of victims could potentially change the level of support for consecutive sentences here observed.

Future research should more deeply examine the role of alternative sanctions. Our experiments used one alternative sanction (parole) as a way to test deterrence motivations (E1) or to rule them out (E2 and E3). In E1, we also examined sentencing adjustments in response to rehabilitative services. From these design features, we demonstrated that the pattern of mitigation across consecutive and concurrent conditions was robust to such factors. Future research should also consider directly testing attitudes about consecutive sentences relative to rehabilitative sanctions. Rehabilitation is both interesting and complicated because, theoretically, it can satisfy multiple punishment motives, including deterrence and retribution. It is possible that support for consecutive life sentences could decrease when faced by such alternatives, but more work would be needed to discern the motivations driving such support.

Our pattern of results is not easily explained as merely a preference to grant professional judges discretion in sentencing. We found, for instance, that people did not support the parole option (E1 and E2) and that proponents of consecutive life sentences were less satisfied than opponents by the prospect of serious offenders are given only one life sentence (E2). These patterns reflect affirmative support for consecutive sentencing per se, though additional research could verify this interpretation by ruling out any role for judicial discretion in the ballot narrative.

Finally, associations between support for consecutive life sentencing and basic demographic variables (age, gender, and socio-economic status) were absent or inconsistent across experiments. An in-depth analysis of demographic differences was beyond the scope of this project, but extensions of this research should consider how such factors might help to shape sentencing attitudes.

Limitations notwithstanding, the punishment behavior of our participants demands explanation. Why did the participants in our study cling to a pattern of punishment that has no direct consequentialist value? Such punishments might carry symbolic value, but, if not to achieve some material consequence, what might they symbolize? At a proximate level of explanation, it could be that these punishments reflect metaphysical beliefs about punishment in the afterlife (see [65]), but our question assessing such beliefs (E2) does not support this interpretation. Another possibility is that these punishments reflect a retributive motivation to represent each life taken, perhaps believing that when there are multiple victims, each victim requires their own unique (not concurrent) sentence. However, results of E2 show that people supported longer-than-life sentences at least as strongly in response to multiple crimes against a single victim as they did in response to crimes against multiple victims (E3). Testing support for multiple death sentences, such as for an offender with multiple victims [66], though rare, could offer another way to examine the generality of this response, as compared to a single death sentence. Unless we appeal to a heuristic explanation, it remains unclear why such offenses could not be adequately represented by a single life (or death) sentence, given that the actual outcome for the offender is the same, as are the outcomes that members of society observe.

Understanding these implicit motivations would benefit from consideration of the potential ultimate functions of punishment. One possibility, consistent with heuristic reasoning, is that, in ancestral environments where punishing too little tended to evoke greater retaliation than punishing too much, more punitive strategies would have had a fitness advantage, even in situations where it might not be strictly necessary. This interpretation aligns with other research on punishment in one-shot economic games [18,67,68]. Another possibility is that over-punishing signals a total, but honest, commitment to winning [19], which could be a more effective deterrent than more conservative strategies.

A third possibility is that our punishment psychology evolved to track and communicate social credits and debits among victim and offender coalitions. Registering these social credits and debits into public memory could serve to maintain reciprocity among both parties [69,70]. So, a preference for longer-than-life punishments could be an expression of this unconscious tendency to use punishment as a public registry. This theory could help to explain other findings involving displaced aggression (e.g., [71]) and parochial altruism (e.g., [72]). This theory could be tested by independently varying whether punishments are disclosed to victims, offenders, and/or other stakeholders, and by observing its effects on memory for, and satisfaction with, the punishment, and on subsequent cooperation behavior. If punishment psychology works as a social accounting ledger, such a system might confer utility, even today. This possibility raises interesting questions about the extent to which longer-than-life punishers are fulfilling the core functions of the justice system or over-generalizing unconscious cognitive rules, or both. (These two alternatives are not necessarily rivalrous. The modern philosophy of “rule utilitarianism”, for instance, posits that an act’s social utility ought to be estimated with respect to whether it conforms to specific normative rules because it is impractical to evaluate the utility of particular acts with respect to a more general utilitarian rule. As a result, rule utilitarianism over-generalizes the rules, but this is by design [73].) We hope our study results motivate further research on this question, and on the amenability of heuristic punishment to modification by competing goals and consequences.

If punishment attitudes and judgments are driven by heuristic reasoning processes, is this necessarily a bad thing? As one of our own participants suggested, even if longer-than-life punishments appear to overgeneralize the rule, there is no material cost to doing this, so no justification for this behavior is needed. From the strictest consequentialist perspective, this might be true. There is no obvious harm in allowing consecutive life sentences. And participants in our voting study appeared to be insensitive to the implementation costs that we specified. Still, it is not clear that longer-than-life sentences are entirely cost-free. First, new policies often do introduce costs. And previous research has shown that, though people tend to discount sentencing costs and other negative consequences when forming punishment attitudes, they often factor these costs into their sentencing judgments (in the form of a sentencing reduction) when those costs are made more salient to them (e.g., [74]). This conditional response to sentencing costs could not be fully explained as a rational economic response to new information, since it occurs even among professional judges and prosecutors who are presumably already familiar with these various costs [75,76]. Instead, this effect has been explained as a heuristic reasoning process (e.g., [77]). One policy implication of such behavior is that the acute salience of sentencing cost–benefit information is likely to have a predictable impact on people’s support for incarceration. Second, scholars have argued that legal doctrines that are out of step with citizens’ values could reduce trust in, and respect for, the justice system [39]; see also, [78]), and over a quarter of our participants agreed with this concern. Policymakers, thus, would benefit from understanding which decision factors modulate public support for such policies. Finally, the special case of consecutive life sentences might be prognostic of irrational punishment behavior in other, less innocuous cases, such as the roughly 500,000 *nonviolent* offenders who are currently incarcerated in the U.S., who might be better served through probation, or the increasing use of life without parole sentences, issued even for nonviolent crimes by repeat offenders. Arguably, these offenders, too, are often being detained for reasons that are largely symbolic rather than pragmatic. Are these sanctions costly enough to outweigh the benefits of symbolic punishment? That is an important normative question that empirical punishment research can help to elucidate.

The social sciences are replete with evidence that people are limited in their awareness of the causes of their behavior (see [79]) and in the ability to predict their own preferences (e.g., [80]). Extending this theme, our study can help people, including voters and legislators, understand and anticipate lay policy preferences about criminal sentencing, as well as some of the factors likely to influence those preferences.

## Figures and Tables

**Table 1:** Effects of sentence type and reduction context on sentence length.

Group	Variance	*SD*
Random effects:		
ID	256.13	16.00
Residual	12.72	3.566
	Coefficient (SE)	*t*-value (*p*)
Fixed effects:		
Intercept	28.87 (2.06)	14.00 (<0.001)
Consecutive baseline	−9.55 (2.89)	−3.30 (0.001)
Reduction^1^ (linear)	−5.55 (0.46)	−12.14 (<0.001)
Reduction^2^ (quadratic)	1.45 (0.46)	3.18 (0.002)
Reduction^3^ (cubic)	0.32 (0.46)	0.69 (0.489)
Reduction^4^ (quartic)	−0.31 (0.46)	−0.69 (0.493)
Consecutive × Reduction^1^	0.85 (0.64)	1.32 (0.186)
Consecutive × Reduction^2^	−0.13 (0.64)	−0.19 (0.846)
Consecutive × Reduction^3^	−0.17 (0.64)	−0.26 (0.795)
Consecutive × Reduction^4^	0.50 (0.64)	0.78 (0.437)

*Note.* Results of robust linear mixed-effects model testing the association between sentence type (Consecutive or Concurrent, where Concurrent serves as the reference class) and reduction context (Baseline, Rehabilitation, Brain damage, Unfair, or Trust) as fixed effects, and sentence length (measured in years), with participant identifier as a random effect. Polynomial relationships show a significant negative linear effect and a positive quadratic effect.

**Table 2:** Self-reported agreement with manipulation check statements.

	One-Sample *t*-Test				Independent Samples *t*-Test				
	*M* (*SD*)	*t*-Value (*p*)	95% CI	*d*	Concurrent *M* (*SD*)	Consecutive *M* (*SD*)	*t*-Value (*p*)	95% CI	*d*
Mr. Smith deeply regrets the harm he caused.	0.42 (1.44)	3.36 (<0.001)	0.17, 0.67	0.30	0.44 (1.39)	0.41 (1.49)	0.11 (0.455)	−0.47, 0.53	--
Mr. Smith has difficulty controlling his impulses.	1.23 (1.10)	12.72 (<0.001)	1.04, 1.42	1.12	1.19 (1.18)	1.27 (1.03)	−0.44 (0.331)	−0.47, 0.30	--
It would be unfair to Mr. Smith if other offenders received lighter sentences for more harmful crimes than he committed.	0.40 (1.76)	2.59 (0.005)	0.09, 0.71	0.23	0.61 (1.74)	0.20 (1.77)	−0.44 (0.331)	−0.20, 1.02	--
Imposing extremely long sentences could reduce the public’s trust in the justice system if these sentences are usually longer than the actual time served.	0.18 (1.59)	1.33 (0.094)	−0.09, 0.46	0.12	0.23 (1.67)	0.14 (1.52)	0.35 (0.363)	−0.46, 0.65	--
I would feel satisfied if Mr. Smith was given only one 50-year prison sentence.	−0.11 (1.95)	−0.63 (0.265)	−0.45, 0.23	--	0.64 (1.85)	−0.83 (1.78)	4.64 (<0.001)	0.85, 2.10	0.81
Judges should be allowed to impose prison sentences that exceed the human lifespan.	0.81 (1.69)	5.44 (<0.001)	0.51, 1.10	0.48	0.70 (1.77)	0.91 (1.62)	−0.69 (0.245)	−0.80, 0.38	--
Judges should be allowed to impose multiple sentences consecutively, meaning that the offender serves them one after the other.	1.25 (1.45)	9.87 (<0.001)	1.00, 1.51	0.87	1.17 (1.54)	1.33 (1.36)	−0.63 (0.264)	−0.67, 0.34	--
Judges should be allowed to impose multiple sentences concurrently, meaning that the offender serves them at the same time.	0.20 (1.68)	1.36 (0.089)	−0.09, 0.49	0.12	0.41 (1.68)	0.0 (1.67)	1.38 (0.085)	−0.18, 0.99	0.24

*Note.* Left: results of one-sample *t*-tests (*df* = 129) assessing agreement or disagreement with each statement relative to the scale midpoint (“neither agree nor disagree”). Right: results of independent samples *t*-tests (*df* = 128) assessing differences in agreement between experimental conditions. Effect size reported as Cohen’s *d*, where significant.

**Table 3:** Percentage of stated punishment justifications within and between conditions.

	Concurrent Group (%)	Consecutive Group (%)	Whole Sample (%)
Retributive	64.1	43.9	53.8
Consequentialist	9.4	16.7	13.1
Communicative	0.0	4.5	2.3
Arbitrary	6.3	19.7	13.1
Unjustified assertion	18.8	22.7	20.8
Negative reason	9.4	9.1	10.0

*Note*: Percentage of participants in the concurrent condition (N = 64), the consecutive condition (N = 66), and the whole sample giving retributive, consequentialist, communicative and other justifications for their punishment recommendations, as judged by at least one of the two trained raters.

## Data Availability

The original data and supporting materials for this study are openly available on Open Science Framework at http://doi.org/10.17605/OSF.IO/YKMNV (accessed on 21 August 2024).

## Supplementary Materials

The following supporting information can be downloaded at: https://www.mdpi.com/article/10.3390/bs14090855/s1, Section S1: Study Materials; Section S2: Supplemental Analyses; Figure S1: Sentence clustering: Total sample; Figure S2: Sentence clustering: Concurrent condition; Figure S3. Sentence clustering: Consecutive condition; Figure S4: Percentage of participants in each condition who voted for or against the consecutive life sentence policy, showing disproportionate preference for consecutive sentences regardless of how the choice was framed; Figure S5: Percentage of participants in each condition who voted for or against the consecutive life sentence policy, showing disproportionate preference for consecutive sentences regardless of implementation costs; Table S1: Comparison between punishment justifications within each condition.
