# Supplementary material for: Punishment after Life: How Attitudes about Longer-than-Life Sentences Expose the Rules of Retribution
Source: Behav Sci (Basel). 2024 Sep 23;14(9):855. doi: 10.3390/bs14090855 (PMC11429324; doi:10.3390/bs14090855)
Supplement: Supplementary file 1 [file behavsci-14-00855-s001.zip › behavsci-3209698-supplementary.pdf]

Supplementary Material

Section S1: Study Materials

Materials: Experiment 1 (E1)

Case summary:

Joe Smith is a 50-year-old male recently convicted of the attempted murder of two young children. After being fired from his job as a receptionist at a pediatrician's office, Mr. Smith entered the office and began shooting at two children in the waiting room. His first shot struck a child in the spine, making her paralyzed. In a second attempt to fire, his gun jammed. At that time, he lunged toward the two children, hitting one in the skull with his gun and stomping on another, breaking several of the child's bones before being apprehended by a security guard. His conviction was based on video surveillance, a witness, and a confession.

Manipulation:

Since Mr. Smith had two victims, he qualifies for two sentences, each with a maximum of 50 years. In your jurisdiction, when an offender qualifies for two sentences that exceed a natural lifespan, those sentences are treated as [*concurrent / consecutive*], meaning that they are served [at the same time / one after the other]. As a result, the combined maximum sentence Mr. Smith can receive is [50 / 100] years in prison. The minimum prison sentence he can receive is [10 / 60] years plus parole. Based on his age, there is a 99.9% chance that Mr. Smith will die within 50 yrs.

Baseline sentencing measure:

[DV 1] The minimum possible sentence that Mr. Smith can receive is [10 / 60] years in prison plus parole. Therefore, he could [not] possibly be released in his lifetime. For how many total years should Mr. Smith be sentenced in prison?

[Concurrent scale:] 10 - 50 yrs.
[Consecutive scale:] 60 - 100 yrs.

Mitigating factors:

In the sentencing hearing, Mr. Smith's attorney was permitted to present evidence of mitigating circumstances. On the following pages, you will read about the attorney's reasons why Mr. Smith's sentence should be reduced. The maximum reduction allowed is 40 years.

[Rehabilitation] Exhibit A. His attorney provided evidence that Mr. Smith has successfully completed extensive therapy in the jail where he awaited trial over the past two years. Two independent therapists testified that Mr. Smith has come to understand and deeply regret the harm he caused to his victims and their families. He has been donating all of his earnings from the job he has been doing in the jail to the victims' families, and has asked to be able to talk with them to express his remorse and apologies.

[DV 2.1] Based on this new evidence, you now have the option of changing your previously recommended sentence or keeping it the same. Your previous sentence was: [XX.X] years. To change your previous sentence recommendation, drag the cursor from its last position. Otherwise, you can keep it the same.

[Brain damage] Exhibit B. His attorney provided evidence that as a child, Mr. Smith suffered brain damage from a car accident. The damage affected the parts of his brain that control violent impulses, and tests suggest that Mr. Smith has much less control over such impulses than the average person.

[DV 2.2]

[Unfairness] Exhibit C. His attorney provided evidence that many other offenders have received lighter sentences for more harmful crimes. For instance, two offenders each received a shorter sentence for a recent bus bombing that killed 12 people.

[DV 2.3]

[Trust] Exhibit D. His attorney provided evidence that imposing lengthy sentences could reduce the public's trust in the justice system because these sentences are almost always longer than the actual time served. As a result, these lengthy sentences become empty promises.

[DV 2.4]

Materials: Experiment 2 (E2)

MEASURE TO [ALLOW VS. PROHIBIT] CONSECUTIVE LIFE SENTENCES

The proposed bill would [allow judges to order VS. prohibit judges from ordering] consecutive life sentences for individuals convicted of murder and additional serious crimes against the same victim. Consecutive sentences are sentences that are served one after the other; in effect, they add up. Notably, when one of the sentences is life without parole for murder, an additional sentence for a second crime, such as rape, cannot actually be served because it would exceed the offender's lifespan (which is already spent serving the murder sentence). The existing policy, therefore, does not allow for sentences to exceed the offender's lifespan. However, the proposed bill would change that, so instead the public record would show that the two sentences were ordered to be served consecutively.

Under this proposed bill, when an offender is convicted of first-degree murder and another serious crime, such as rape, against the same victim, the judge will be [permitted to order the offender's sentences consecutively, even though their total could exceed the offender’s lifespan. If the judge does not order the sentences consecutively, the sentences will instead be served concurrently, meaning at the same time VS. required to order the offender's sentences concurrently, meaning at the same time. The judge will not be able to choose to order the life sentences consecutively, so their total could exceed the human lifespan.] In either case, the offender would never be eligible for release in his lifetime.

Fiscal Impact. [To enact this policy, there will be no financial burden to the state or its taxpayers VS. The total cost to enact this policy will be *10 Million dollars* to revise existing legal documents and train judges across the state. This cost will be paid evenly by the taxpayers in your State in the form of a new tax.]

A *YES* VOTE would [allow judges to order VS prohibit judges from ordering] consecutive (one after another) sentences or the already-allowed concurrent (at the same time) sentences for individuals convicted of murder and additional serious crimes against the same victim.

A *NO* VOTE would continue to [require judges to order such life sentences to be served concurrently only (at the same time) VS. allow judges to order such life sentences to be served concurrently or consecutively.]

Materials: Supplemental Experiment 1 (E3)

MEASURE TO [ALLOW VS. PROHIBIT] CONSECUTIVE LIFE SENTENCES

The proposed bill would [allow judges to order VS. prohibit judges from ordering] consecutive life sentences for individuals convicted of capital crimes, such as intentional homicide, with multiple victims. Consecutive life sentences are multiple life sentences that are served one after the other. So if one offender were given two life sentences, he would only live long enough to serve one of those two sentences. However, the public record would still show two life sentences, not just the time served.

According to this proposed bill, when an offender is convicted of a capital crime with multiple victims, the judge will be [allowed to order the offender's sentences consecutively, even if their total far exceeds the human lifespan. If the judge does not order the sentences consecutively, the sentences will instead be served concurrently, meaning at the same time VS. required to order the offender's sentences concurrently, meaning at the same time. The judge will not be able to choose to order the life sentences consecutively, so their total cannot exceed the human lifespan.] In either case, the offender would never be eligible for release in his lifetime.

Fiscal Impact. [To change this policy, there will be no financial burden to the state or its taxpayers VS. The total cost to change this policy will be *10 Million dollars* to revise existing legal documents and train judges across the state. This cost will be paid evenly by the taxpayers in your State in the form of a new tax.]

A *YES* VOTE would [allow judges to order VS prohibit judges from ordering] multiple life sentences to be served concurrently or consecutively for individuals convicted of capital crimes with multiple victims.

A *NO* VOTE would continue to [require judges to order such life sentences to be served concurrently only VS. allow judges to order such life sentences to be served concurrently or consecutively.]

Section S2: Supplemental Analyses

Experiment 1: Analysis of punishment justifications

Table S1. Comparison between punishment justifications within each condition

**Whole Sample**	(1)<br>*t*-value (*p*)<br>Cohen's *d*	(2)<br>*t*-value (*p*)<br>Cohen's *d*	(3)<br>*t*-value (*p*)<br>Cohen's *d*	(4)<br>*t*-value (*p*)<br>Cohen's *d*	(5)<br>*t*-value (*p*)<br>Cohen's *d*
(1) Retribution	--	--	--	--	--
(2) Consequentialism	7.17 $^{(< .001)}$<br>.63	--	--	--	--
(3) Communication	11.50 $^{(< .001)}$<br>1.01	3.60 $^{(< .001)}$<br>.32	--	--	--
(4) Arbitrary	6.63 $^{(< .001)}$<br>.58	-.09 $^{(.931)}$<br>--	-3.44 $^{(< .001)}$<br>-.30	--	--
(5) Unjustified assertion	4.79 $^{(< .001)}$<br>.42	-1.54 $^{(.126)}$<br>--	-4.87 $^{(< .001)}$ -<br>4.23	1.45 $^{(.149)}$<br>--	--
(6) Negative reason	8.37 $^{(< .001)}$<br>.73	.89 $^{(.377)}$<br>--	-2.62 $^{(.010)}$ -<br>.23	.94 $^{(.351)}$<br>--	2.53 $^{(.013)}$<br>.22

**Concurrent condition**	(1)<br>*t*-value (*p*)<br>Cohen's *d*	(2)<br>*t*-value (*p*)<br>Cohen's *d*	(3)<br>*t*-value (*p*)<br>Cohen's *d*	(4)<br>*t*-value (*p*)<br>Cohen's *d*	(5)<br>*t*-value (*p*)<br>Cohen's *d*
(1) Retribution	--	--	--	--	--
(2) Consequentialism	7.26 $^{(< .001)}$<br>.91	--	--	--	--
(3) Communication	10.60 $^{(< .001)}$<br>1.33	2.50 $^{(.015)}$<br>.31	--	--	--
(4) Arbitrary	7.56 $^{(< .001)}$<br>.94	.49 $^{(.626)}$<br>--	-2.05 $^{(.045)}$<br>-.26	--	--

(5) Unjustified assertion	4.71 $^{(< .001)}$<br>.59	-1.49 $^{(.141)}$<br>--	-3.77 $^{(< .001)}$<br>-4.71	1.96 $^{(.054)}$<br>--	--
(6) Negative reason	7.42 $^{(< .001)}$<br>.93	.00 $^{(1.000)}$<br>--	-2.50 $^{(.015)}$<br>-.31	-.49 $^{(.626)}$<br>--	1.52 $^{(.135)}$<br>--

**Consecutive condition**	(1)<br>*t*-value (*p*)<br>Cohen's *d*	(2)<br>*t*-value (*p*)<br>Cohen's *d*	(3)<br>*t*-value (*p*)<br>Cohen's *d*	(4)<br>*t*-value (*p*)<br>Cohen's *d*	(5)<br>*t*-value (*p*)<br>Cohen's *d*
(1) Retribution	--	--	--	--	--
(2) Consequentialism	3.31 $^{(.002)}$<br>.41	--	--	--	--
(3) Communication	6.27 $^{(< .001)}$<br>.77	2.65 $^{(.010)}$<br>.33	--	--	--
(4) Arbitrary	2.64 $^{(.010)}$<br>.33	-.41 $^{(.686)}$<br>--	-2.83 $^{(.006)}$<br>-.35	--	--
(5) Unjustified assertion	2.17 $^{(.034)}$<br>.27	-.78 $^{(.437)}$<br>--	-3.22 $^{(.002)}$<br>-.40	.38 $^{(.709)}$<br>--	--
(6) Negative reason	4.60 $^{(< .001)}$<br>.57	1.16 $^{(.252)}$<br>--	-1.38 $^{(.172)}$<br>--	1.50 $^{(.140)}$<br>--	2.04 $^{(.046)}$<br>.25

Note: **p* < .05, ***p* < .01, ****p* < .001, *df* = 129. All *t*-values derive from two-tailed paired-samples *t*-tests comparing group means. Effect size reported as Cohen's *d*, where significant.

To further explore why participants might favor longer-than-life sentences, we assessed, using a structured question, the association between participants' sentencing behavior and their philosophical justifications for punishment in general (retributive vs. consequentialist). Because justification scores followed a bimodal distribution, they were dummy-coded for use in a point-biserial correlation analysis. Participants' justifications were not associated with baseline sentencing recommendations or with sentencing reductions (defined as the mean of the four sentencing recommendations) in the concurrent condition (baseline: $r(55) = 0.10$, $p = .453$; reduction: $r(55) = 0.18$, $p = .177$) or the consecutive condition (baseline: $r(60) = 0.12$, $p = .354$; reduction: $r(60) = 0.24$, $p = .057$).

Experiment 1: Analysis of clustering effects in sentencing scores

Previous research has discovered evidence of context effects on sentencing in the form of rounding to the nearest conceptual anchor, such as increments of five or ten (e.g., Jones & Rankin, 2014). To assess the presence of rounding in our sentencing recommendations, we first observed, through visual inspection of our sentencing distributions, apparent clustering at increments of five. We then performed a Two-step Cluster Analysis to test dispersion in a nine-cluster model corresponding to the nine multiples of five within our scale with a range of 40 years. The average silhouette score was 1.0 out of 1.0, indicating significant clustering. Visual inspection confirms a nine-cluster solution with each mode centered on a multiple of five. (See Figures S1 - S3 below.) As scholars have argued, such round effects are common in legal data despite there being no known legal justification for it, suggesting the operation of heuristic reasoning (Jones & Rankin, 2014).

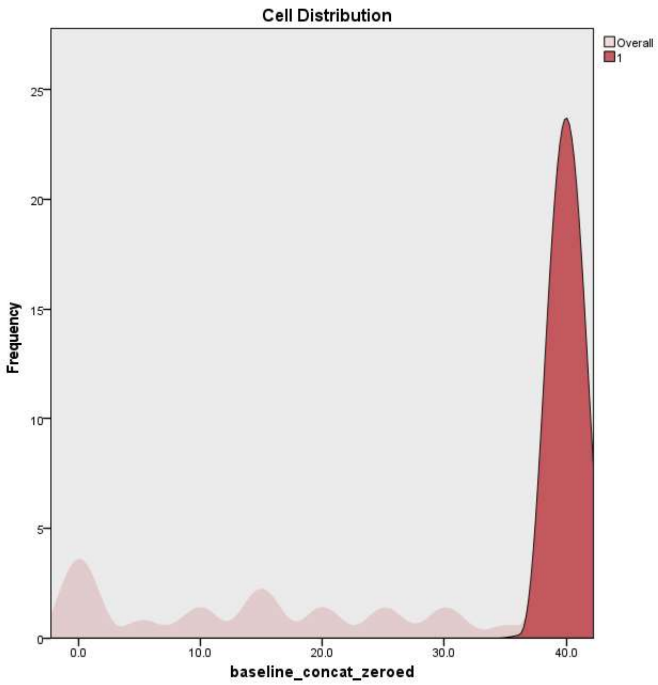

Figure S1. Sentence clustering: Total sample.

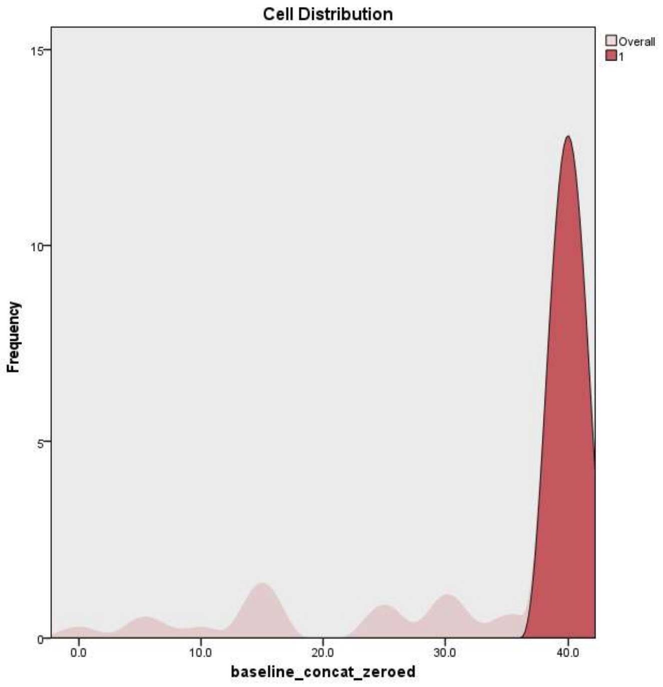

Figure S2. Sentence clustering: Concurrent condition.

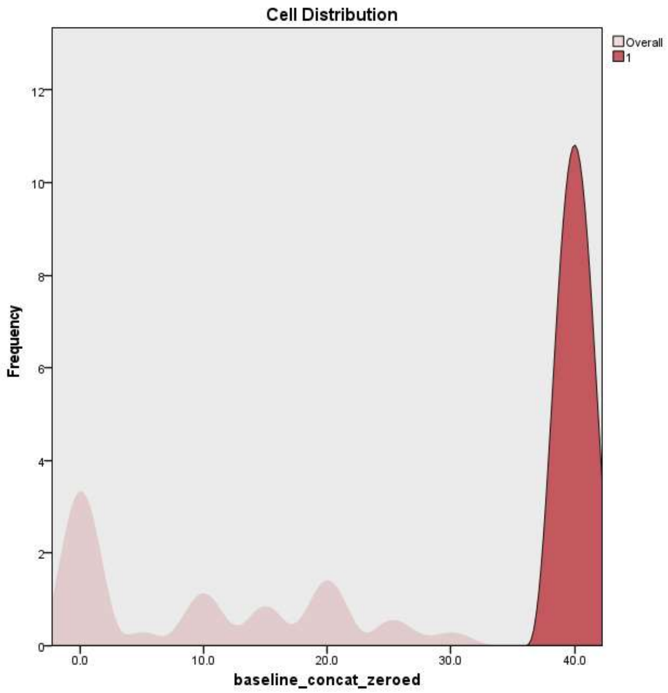

Figure S3. Sentence clustering: Consecutive condition.

Experiment 2: Analysis of policy support and abstract philosophical justifications for punishment

Although voting choices are typically presented in discrete formats (e.g., yes or no), we also assessed support for the consecutive life sentencing policy in a continuous format (the 7-point scale asking the degree of (dis)agreement with the voting decision) because of the greater statistical sensitivity that such measures afford. Overall support for the consecutive life sentencing policy (on the continuous scale) was significantly greater than zero, $t(181) = 6.02$, two-tailed $p < .001$, 95% CI [0.59, 1.16], $d = 0.45$, consistent with our other findings. Next, we tested the effects of our two independent variables on this continuous measure of support. Using a two-way Analysis of Variance, the overall model was significant, $F(3, 178) = 6.69$, $p < .001$. This effect was driven by an antagonistic interaction, $F(1, 178) = 19.23$, $p < .001$, whereby people showed more support under the "allow" frame ($M = 1.61$, $SE = 0.27$, 95% CI[1.09, 2.14]) than the "prohibit" frame ($M = 0.25$, $SE = 0.28$, 95% CI[-0.31, 0.81]), but only when the cost was low. When the cost was high, they showed more support under the "prohibit" frame ($M = 1.32$, $SE = 0.28$, 95% CI[0.76, 1.88]) than the "allow" frame ($M = 0.24$, $SE = 0.28$, 95% CI[-0.31, 0.80]). There were no main effects for the default frame, $F(1, 178) = 0.27$, $p = .60$, or the cost manipulation, $F(1, 178) = 0.29$, $p = .59$. This is a novel finding, but the pattern is consistent with established theories, such as Prospect Theory's explanation of loss aversion, namely the phenomenon that people tend to value avoiding losses more than obtaining gains (Tversky & Kahneman, 1992). As with E2, support for consecutive life sentences was not associated with age, $r(139) = .00$, $p = .997$, or socioeconomic status, $r(182) = 0.02$, $p = .835$. It was also not associated with gender, $r(182) = -0.08$, $p = .266$, or political ideology, $r(180) = 0.14$, $p = .059$.

As in Experiment 1, we assessed the association between participants' sentencing behavior and their abstract philosophical justifications for punishment (using a structured question) in an attempt to explain why participants might favor consecutive life sentences. This time, instead of limiting their choices to retributive vs. consequentialist justifications, we asked them to rank their agreement with four different justifications: retributive, consequentialist, rehabilitative, and communicative, then examined the extent to which support for the consecutive life sentencing policy (using the continuous measure of support) correlated with each of these justifications. Support for consecutive life sentencing was positively correlated with endorsement of the consequentialist justification of protecting society from harm, Spearman's $\rho = -0.18$, $p = .014$, and negatively correlated with endorsement of the rehabilitative justification, $\rho = 0.29$, $p < .001$, but not associated with endorsement of the retributive justification of deserts, $\rho = -0.14$, $p = .094$, or the communicative justification to send the offender a message, $\rho = 0.03$, $p = .713$.[1]

Experiment 3: Full analysis

[1] Signs may be flipped because of reverse-scored variables.

A plausible explanation for the heuristic leading people to support consecutive life sentences, even if only symbolic years beyond the lifespan, is that people feel that each victim of a violent crime (or their family) should be recognized with "their own" sentence that does not overlap with others. This explanation for the proximate psychological mechanism driving our effects is consistent with both retributive and communicative punitive motives and predicts that additional victims will elicit stronger support for consecutive life sentences. To examine this prediction, we developed a replication and extension of E2. In this experiment, we tested the degree to which participants might favor consecutive life sentences for serious crimes against multiple victims (as opposed to multiple crimes against one victim) relative to concurrent life sentences.

Method

Participants

Participants were 367 undergraduates recruited using the same procedure as Experiment 1. Fifty-nine were excluded for incomplete data; two for failing a multiple-choice attention check; 15 for failing the catch question instructing to skip the question; three for reporting an age less than 18; and 28 were excluded for failing to recognize the correct definitions of "concurrent" and "consecutive" sentences (i.e., life sentences that are served "one after another" or "at the same time"). This question was included as a comprehension check to be sure that participants understood the relatively technical language presented. The remaining 260 participants (our final sample) reportedly were 61.9% female, 34.6% male, and 7.0% other or unanswered; 13.8% Hispanic or Latino; 22.3% White/Caucasian, 42.3% Black or African American, 24.2% Asian, and 6.6% other or unanswered (ethnic and racial categories were non-exclusive); and with a mean age of 20.25 years ($SD = 5.30$).

A minimum sample size estimate was based on an *a priori* power analysis, using a Chi-square test of independence with one degree of freedom to compare frequencies of vote types as a function of our two manipulations, assuming a medium effect size ($w = 0.20$), which was the smallest effect we were interested in detecting. Assuming an alpha threshold of .05, and a power threshold of 0.80, 197 participants would be sufficient to detect an effect. We sought to exceed this number to account for possible attrition and data exclusion.

Design and Materials

The design structure was the same as E2, which independently varied the default choice frame and implementation cost of the bill. This time, we characterized the legislation in terms of sentencing offenders convicted of a serious crime against *multiple* victims. This study was conducted prior to E2 and served as a pilot study. The follow-up questions were overlapping with E2, but with the following exceptions: A validation question was also included, asking whether judges should be allowed to impose consecutive and/or concurrent life sentences; Our question probing the communicative function of longer-than-life sentences asked, in the form of an ordinal scale rather than a ranking task, whether such sentences send a stronger message about the wrongfulness of the crime; beliefs about the afterlife were not assessed; some of the wording

of the legislation and follow-up questions varied between experiments. Manipulation checks for the default choice frame and implementation cost were not included. All other design features, procedures, measures, and predictions matched those of E2. (See S1 for stimuli.) For E3 preregistration protocol, see [DOI omitted for blind review].

Results

Voting Behavior

Consistent with E2, participants largely favored the option of consecutive life sentences, with 65% voting to allow and just 35% voting to prohibit, $\chi^2(1, 260) = 23.40$, $p < .001$, $w = 0.30$. Like E2, this effect was not detectably influenced by the default choice frame, $\chi^2(1, 260) = 0.08$, $p = .78$, or implementation cost, $\chi^2(1, 260) = 1.53$, $p = .22$ (See Fig. S4 & S5). These findings validate our E2 findings and extend them to attitudes about crimes against multiple victims.

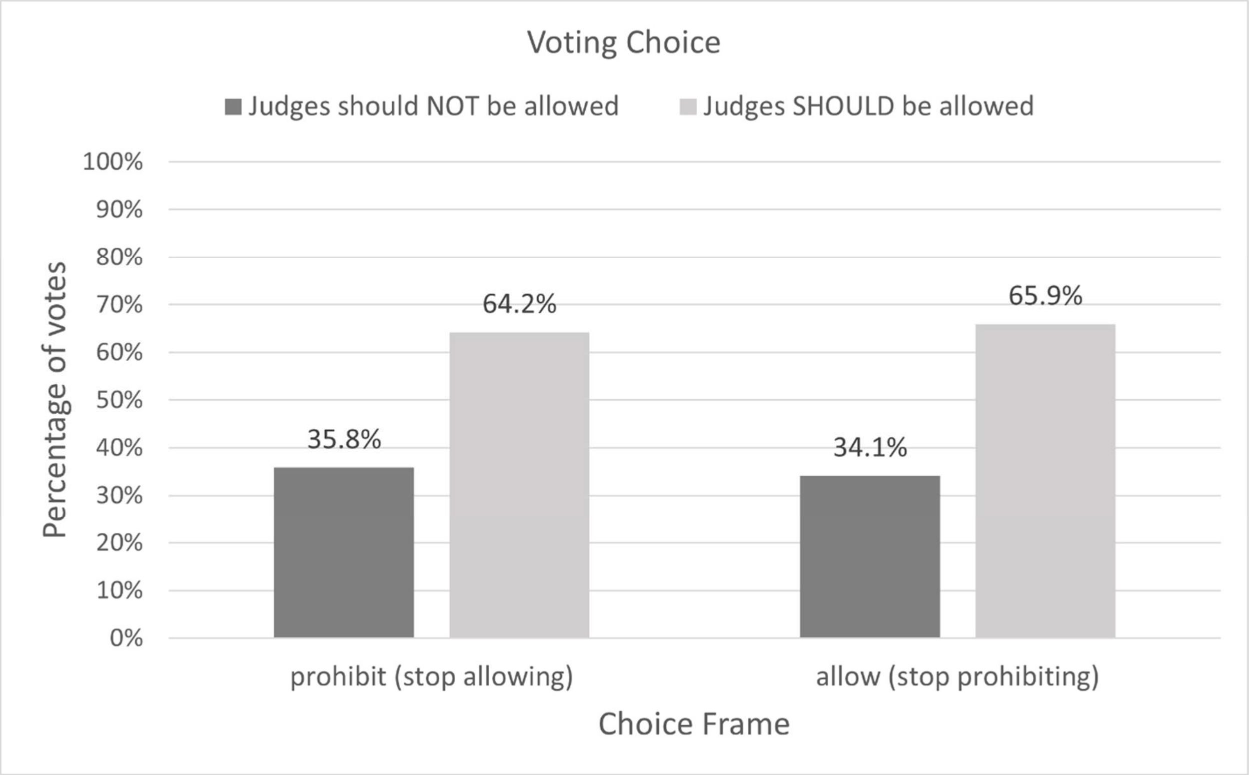

Figure S4. Percentage of participants in each condition who voted for or against the consecutive life sentence policy, showing disproportionate preference for consecutive sentences regardless of how the choice was framed.

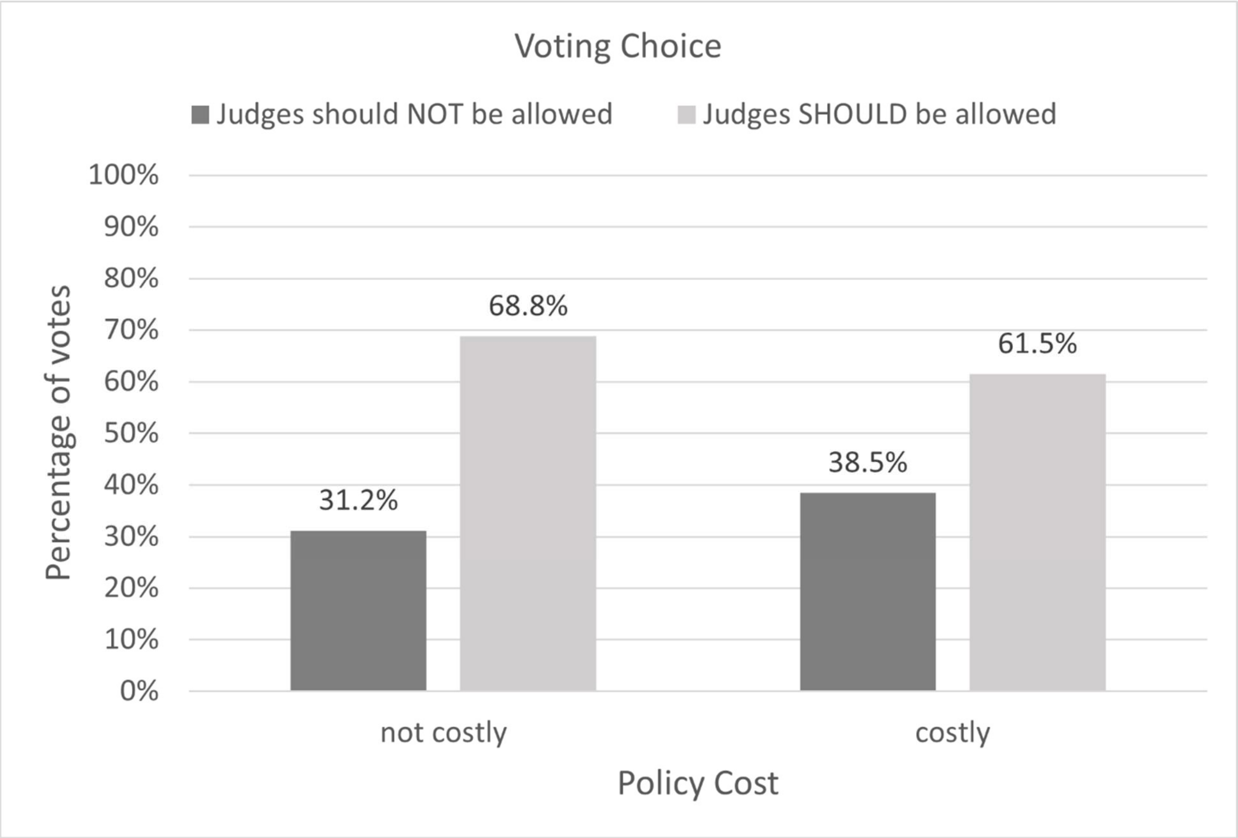

Figure S5. Percentage of participants in each condition who voted for or against the consecutive life sentence policy, showing disproportionate preference for consecutive sentences regardless of implementation costs.

Follow-up Questions

Our follow-up questions confirmed that participants who voted for consecutive life sentences agreed (*M* = 1.36, *SD* = 1.3, 95% CI [1.16, 1.56]) more strongly than their counterparts (*M* = -0.24, *SD* = 1.39, 95% CI [-0.51, .03]) that judges should be allowed to impose consecutive life sentences, $F(1, 258) = 89.98$, $p < .001$, $\eta_p^2 = .259$. Likewise, they were less satisfied (*M* = -0.07, *SD* = 1.61, 95% CI [-0.30, 0.17]) than their counterparts (*M* = 0.85, *SD* = 1.38, 95% CI [0.53, 1.16]) by the prospect that serious offenders with multiple victims would only be given one life sentence, $F(1, 258) = 20.84$, $p < .001$, $\eta_p^2 = .075$, replicating E2. Like E2, this pattern emerged despite the broader sample's agreement that offenders who are sentenced to life without parole are "extremely unlikely" to get out of prison in their lifetime; one-sample $t(259) = 20.56$, $p < .001$, $d = 1.28$ (*M* = 1.64, *SD* = 1.29). Indeed, the participants who voted in favor of consecutive life sentences were no less suspicious about the prospect of early release than those who voted against them, $F(1, 258) = .30$, $p = .58$, minimizing the concern that they only supported consecutive life sentences as a means to protect against the possibility of early release. Ratings between the two groups did not differ about whether judges should be allowed to impose concurrent life sentences, $F(1, 258) = .02$, $p = .901$).

To examine how participants justify their support for consecutive life sentences, they were asked how much they agree with a statement that these sentences send a stronger message about the wrongfulness of the crime (a potential communicative function). While the sample as a whole expressed relative agreement that these sentences send this type of message (*M* = 0.93, *SD* = 1.59), $t(259) = 9.49$, $p < .001$, $d = 0.59$, participants who voted in favor of consecutive life sentences were significantly more likely (*M* = 1.19, *SD* = 1.55, 95% CI [0.95, 1.43]) to agree with this communicative function than those who voted against it (*M* = 0.46, *SD* = 1.57, 95% CI [0.14, 0.78]), $F(1, 258) = 12.99$, $p < .001$, ηp2 = .048. This finding suggests that people ascribe communicative value to the use of consecutive life sentences. Still, it is not clear which punishment theory best explains these sentiments since, as mentioned in footnote 1, different communicative theories make different predictions about longer-than-life sentences, and some of these are consistent with the tenets of retributive theory.

For those opposing consecutive life sentences, these individuals (*M* = 0.26, *SD* = 1.37) were significantly more concerned about the impact on the public's trust in the justice system than those who voted in favor (*M* = -0.18, *SD* = 1.53), $F(1, 258) = 5.30$, $p = .022$, $\eta_p^2 = .020$.

Most participants (59.6%) reported support for the option of a sentencing reduction in light of mitigating circumstances (e.g., brain damage), $\chi^2(1, 260) = 9.62$, $p = .002$, but only for consecutive life sentences, and not extending into the range of real life-years (25.4%), $\chi^2(1, 260) = 63.02$, $p < .001$.

As in E2, we explored support for the consecutive life sentencing policy in a continuous format (7-point scale). A one-sample *t*-test revealed that overall support for the measure was significantly greater than zero, $t(259) = 2.27$, two-tailed $p = .024$, 95% CI [0.03, 0.45], $d = 0.14$, replicating our finding from the discrete choice format. Next, we tested the effects of our two independent variables on this continuous measure of support. Using a two-way Analysis of Variance, the overall model was only marginally significant, $F(3, 256) = 2.32$, $p = .076$, $\eta_p^2 = .026$. This effect was driven by the choice frame, which exerted a main effect on support for consecutive life sentences, $F(1, 256) = 4.25$, $p = .040$, such that those given the "allow" choice frame (*M* = 0.47, *SD* = 1.66, 95% CI [0.17, 0.77]) were more supportive than those given the "prohibit" frame (*M* = .04, *SD* = 1.71, 95% CI [-0.25, 0.34], $\eta_p^2 = .016$). There was no detectable effect of implementation cost, $F(1, 256) = 0.05$, $p = .822$. The interaction was not significant, $F(1, 256) = 2.97$, $p = .086$. If anything, this analysis indicates that the choice frame might have a limited effect on continuous measures of support. However, this effect is small and, in the context of our other hypothesis tests, unlikely to generalize to the discrete choice format. Support for consecutive life sentences was not associated with age, $r(194) = .12$, $p = .084$, or socioeconomic status, $r(228) = -0.07$, $p = .284$. It was also not associated with gender, $r(249) = .01$, $p = .931$, or political ideology, $r(217) = .06$, $p = .389$, contrary to Experiment 1.

As in E2, we assessed the association between participants' sentencing behavior (using the continuous measure of support) and their abstract philosophical justifications for punishment (using a structured question). Two significant correlations were found. First, support for the policy was positively correlated with endorsement of the retributive justification, Spearman's ρ =

0.17, $p = .010$. Second, support for the policy was negatively correlated with endorsement of the rehabilitative justification, $\rho = -.20$, $p = .002$. Policy support was not significantly associated with endorsement of the consequentialist justification, $\rho = -.03$, $p = .672$, or the communicative justification, $\rho = 0.07$, $p = .298$.

Last, we tested the association between support for the consecutive life sentencing policy and beliefs in free will. As with E1, support was positively (though more weakly) associated with free will beliefs, $r(258) = .18$, p = .003.

Supplemental References

Tversky, A., & Kahneman, D. (1992). Advances in prospect theory: Cumulative representation of uncertainty. *Journal of Risk and uncertainty*, *5*, 297-323. https://doi.org/10.1007/BF00122574
